# Learning the Regulatory Code of Gene Expression

**DOI:** 10.3389/fmolb.2021.673363

**Published:** 2021-06-10

**Authors:** Jan Zrimec, Filip Buric, Mariia Kokina, Victor Garcia, Aleksej Zelezniak

**Affiliations:** ^1^ Department of Biology and Biological Engineering, Chalmers University of Technology, Gothenburg, Sweden; ^2^ Novo Nordisk Foundation Center for Biosustainability, Technical University of Denmark, Kongens Lyngby, Denmark; ^3^ School of Life Sciences and Facility Management, Zurich University of Applied Sciences, Wädenswil, Switzerland; ^4^ Science for Life Laboratory, Stockholm, Sweden

**Keywords:** gene expression prediction, cis-regulatory grammar, gene regulatory structure, mRNA & protein abundance, chromatin accessibility, regulatory genomics, machine learning, deep neural networks

## Abstract

Data-driven machine learning is the method of choice for predicting molecular phenotypes from nucleotide sequence, modeling gene expression events including protein-DNA binding, chromatin states as well as mRNA and protein levels. Deep neural networks automatically learn informative sequence representations and interpreting them enables us to improve our understanding of the regulatory code governing gene expression. Here, we review the latest developments that apply shallow or deep learning to quantify molecular phenotypes and decode the *cis*-regulatory grammar from prokaryotic and eukaryotic sequencing data. Our approach is to build from the ground up, first focusing on the initiating protein-DNA interactions, then specific coding and non-coding regions, and finally on advances that combine multiple parts of the gene and mRNA regulatory structures, achieving unprecedented performance. We thus provide a quantitative view of gene expression regulation from nucleotide sequence, concluding with an information-centric overview of the central dogma of molecular biology.

## Introduction

Genetic information is stored and encoded in genes that produce an organism’s phenotype by being expressed through multiple biochemical processes into a variety of functional molecules. The central dogma of molecular biology states that genetic information flows from DNA to the phenotypically relevant proteins of an organism in a unidirectional, two-step process: the transcription of DNA into messenger RNA (mRNA) is followed by translation of mRNA into protein (Watson et al., 2008). From these molecular phenotypes, further post-translational processing and cellular metabolism shape and define the observable phenotype of the organism (Nielsen, 2017). Some of the most important processes involved in gene expression are regulated at the nucleotide sequence level, spanning the coding and non-coding regulatory regions adjacent to the gene (Watson et al., 2008; Zrimec et al., 2020). For over a decade, a key trend in the field has thus been to develop computational methods that can process nucleotide sequences and interpret the regulatory code within them, to better understand gene expression and improve quantitative predictions (Segal and Widom, 2009; Levo and Segal, 2014; Li et al., 2019a). These developments are not only important for advancing molecular biology, but have practical implications as well: they are crucial for solving problems related to human disease (Lee and Young, 2013; Zhou et al., 2018a) as well as biotechnology applications (de Jongh et al., 2020).

The key interactions that govern gene expression occur among proteins and nucleic acids. Proteins search for their active binding sites by sliding and diffusion, recognizing a particular DNA site *via* physicochemical interactions with the molecule (Tafvizi et al., 2011; Hammar et al., 2012). Typical binding domains of DNA-binding proteins (DBPs), such as transcription factors (TFs) and polymerases, include helix-turn-helix and zinc finger domains (Watson et al., 2008). However, besides direct protein-DNA readout with the major groove of the DNA helix, which offers base-specific hydrogen bond donors, acceptors, and nonpolar groups that can be recognized by complementary groups on the amino acid side chain, the specificities of protein-DNA interactions are defined also by indirect readout (Rohs et al., 2010; Marcovitz and Levy, 2013; Inukai et al., 2017). This comprises “weak” protein-DNA interactions that depend on base pairs that are not directly contacted by the protein and are defined by conformational and physicochemical DNA properties at the specific binding sites or in their vicinity (Rohs et al., 2009; Yang et al., 2017; Zrimec and Lapanje, 2018). On the other hand, RNA is a single stranded molecule with a softer backbone than DNA and thus has more extensive secondary and tertiary structure. RNA-binding proteins (RBPs) recognize single or double stranded RNA, three-dimensional structural features of folded RNAs, or even bind RNA non-specifically (Re et al., 2014). In regulating translation, however, multiple conserved RNA sequence motifs have been uncovered that play a key role typically *via* single strand or secondary structure-recognition mechanisms (Watson et al., 2008; Leppek et al., 2018). Therefore, despite the apparent monomeric simplicity of nucleic acid sequences, the problem of extracting information from them is quite complex, as they encode a rich grammar of motif occurrences, combinations and sequential properties that needs to be correctly interpreted (Siggers and Gordân, 2014; Slattery et al., 2014; Li et al., 2019a; Nagy and Nagy, 2020).

In this regard, machine learning (ML) comprises a set of algorithms that are capable of mapping complex relationships between input and target variables in a supervised fashion. The resulting predictive/descriptive models can perform classification of discrete target variables or regression of continuous ones. Classical algorithms, which include (multiple) linear regression (LR), support vector machines (SVMs), tree-based methods such as random forests (RFs), and feedforward neural networks (NNs) (Hastie et al., 2013; Géron, 2019), commonly referred to as “shallow” methods, have in recent years been superseded by deep neural networks (DNNs) (LeCun et al., 2015). DNNs resolve many problems inherent to the shallow methods, such as the reliance on feature engineering and selection, but come at the cost of requiring orders of magnitude more training data and computational resources (Angermueller et al., 2016; Eraslan et al., 2019a). In the current big data era, however, this is a diminishing problem. The result is that the information in nucleotide sequences can now be deciphered at unprecedented scale and quality, elucidating the regulatory grammar and greatly expanding our understanding of the underlying processes and capacity to accurately predict the outcomes of gene expression (Zhou et al., 2018a; Eraslan et al., 2019a; Zrimec et al., 2020).

In the present review, we provide an overview of the latest published developments that apply ML to nucleotide sequence data in order to understand gene expression in the most well studied model organisms, including bacteria (*Escherichia coli*), unicellular eukaryotes (yeast, *Saccharomyces cerevisiae*) and multicellular eukaryotes (human, *Homo sapiens*). Since these organisms represent the whole spectrum of genetic regulatory complexity, with gene densities ranging from 892 (bacteria) to six (human) genes per Mbp (Zrimec et al., 2020), the knowledge and principles presented here are generally applicable to all other organisms including insects and plants (Haberle and Stark, 2018; Wang H. et al., 2020). We specifically focus on the latest developments with deep learning and compare them to the state of the art solutions with shallow methods. By reasoning from first principles, the problem of predicting gene expression levels from nucleotide sequence data is explained from the ground up by deconstructing it into the basic regulatory processes and grammatical elements. We first focus on modeling the protein-DNA interactions important for initiating transcription, which include TF binding and nucleosome positioning. We then detail the current understanding of the regulatory grammar carried within the specific coding and non-coding regulatory regions, and its involvement in defining transcript and protein abundance. Based on these principles, we review advanced modeling approaches that use multiple different parts of the gene regulatory structure or whole nucleotide sequences, demonstrating how this increases their predictive power. Finally, by considering all the results, we provide an information-centric overview of the field, and discuss the applicative potential and future outlook of the presented modeling approaches.

## Learning the Protein-DNA Interactions Initiating Gene Expression

**FIGURE 1:**
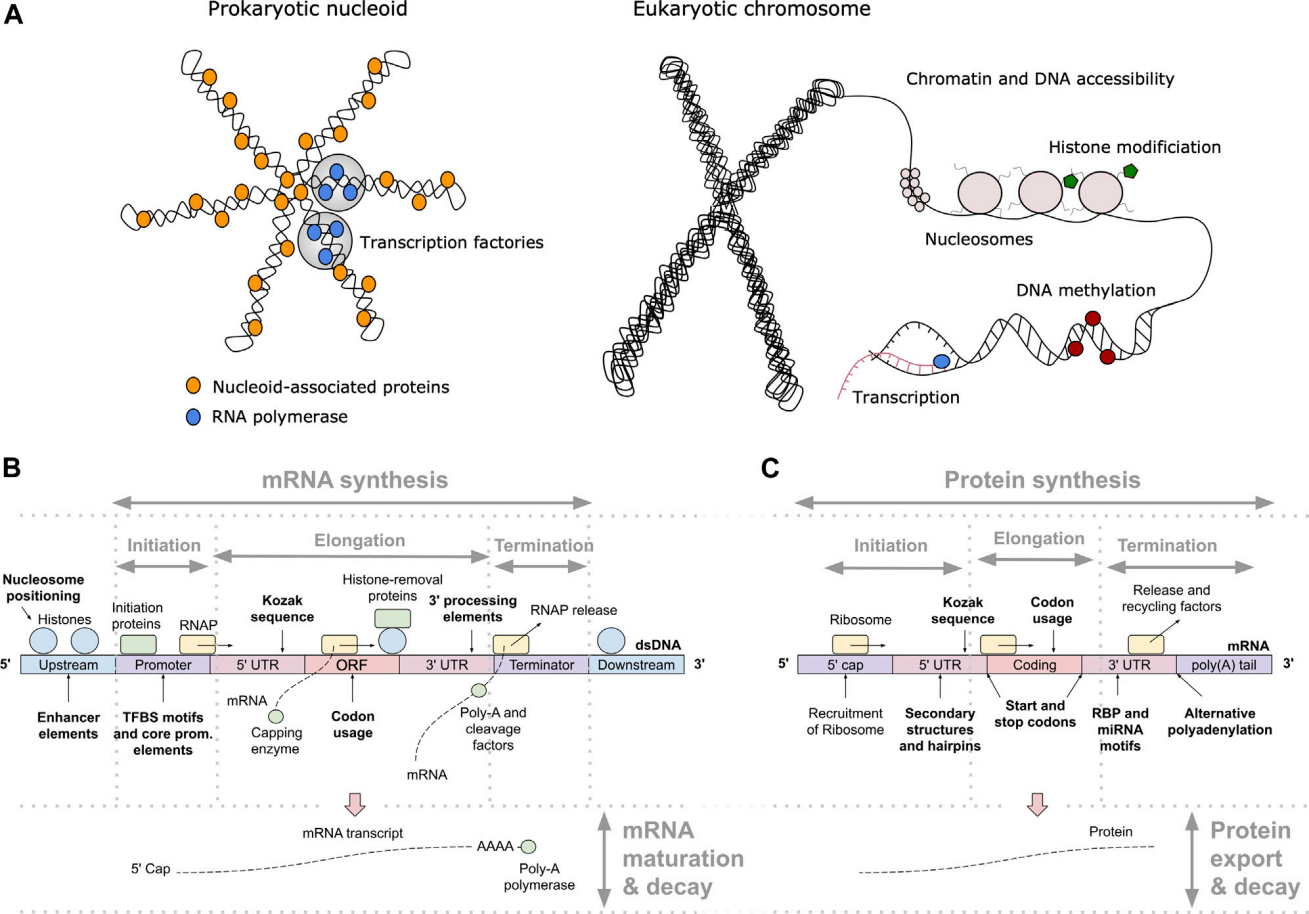
Principles of gene expression. **(A)** Protein-DNA interactions in prokaryotic nucleoid and eukaryotic chromosome structure, epigenetics and transcription initiation. The basic repeating structural unit of chromatin is the nucleosome, which contains eight histone proteins. Bacterial nucleoid-associated proteins are the main regulators of nucleoid structure, where the circular genome is supercoiled and uncoiled by these proteins. In cells, genes are switched on and off based on the need for product in response to cellular and environmental signals. This is regulated predominantly at the level of transcription initiation, where chromatin and nucleoid structure open and close, controlling the accessibility of DNA and defining areas with high amounts of transcription (factories) upon demand. **(B)** Depiction of eukaryotic transcription across the gene regulatory structure that includes coding and non-coding regulatory regions. The open reading frame (ORF) carries the coding sequence, constructed in the process of splicing by joining introns and removing exons. Each region carries specific regulatory signals, including transcription factor binding sites (TFBS) in enhancers, core promoter elements in promoters, Kozak sequence in 5′ untranslated regions (UTRs), codon usage bias of coding regions and multiple termination signals in 3′ UTRs and terminators, which are common predictive features in ML (highlighted bold). RNAP denotes RNA polymerase, mRNA messenger RNA. **(C)** Depiction of eukaryotic translation across the mRNA regulatory structure, where initiation involves the 5′ cap, Kozak sequence and secondary structures in the 5′ UTR. Codon usage bias affects elongation, whereas RNA-binding protein (RBP) sites, microRNA (miRNA) response elements and alternative polyadenylation in the 3′ UTR affect post-translational processing and final expression levels. These regulatory elements are common predictive features in ML (highlighted bold).

One of the key regulation strategies of gene expression is at the level of transcription initiation (Watson et al., 2008), which is also the most studied and modeled regulatory mechanism (Segal and Widom, 2009; Levo and Segal, 2014). Transcription initiation is a complex process involving many different interacting DNA and protein components, including: 1) activating or repressing TFs that bind 6–12 bp long TF binding sites (TFBS) in enhancer and promoter regions (Watson et al., 2008) with different binding affinities and specificities (Levo and Segal, 2014), 2) nucleosomes that form around 147 bp long DNA stretches and define chromatin accessibility, acting as general transcriptional repressors by competing with TFs for DNA binding (Segal and Widom, 2009; Struhl and Segal, 2013), 3) other components of the transcription initiation enzymatic machinery including sigma factors (σ) in prokaryotes and components (TFIID/SAGA, mediator) of the preinitiation complex (PIC) in eukaryotes (Feklístov et al., 2014; Haberle and Stark, 2018), and 4) physicochemical and thermodynamic properties related to protein binding (Rohs et al., 2010; Inukai et al., 2017) and transcription initiation (Chen et al., 2010; Zrimec and Lapanje, 2015), such as strand dissociation around the transcription start site (TSS), giving enzymatic access to the DNA (Figure 1A). The DNA sequence preferences of nucleosomes define nucleosome organization *in vivo* and have been shown to account for the general depletion of nucleosomes around the starts and ends of genes as well as around TFBS, which might assist in directing TFs to their appropriate genomic sites (Segal and Widom, 2009). Apart from the DNA-guided nucleosome positioning, other epigenetic mechanisms (where functionally relevant changes to the genome do not involve a change in the nucleotide sequence), such as histone modification and DNA methylation, also play a vital part in transcriptional regulation (Gibney and Nolan, 2010; Miller and Grant, 2013). Together, they control the accessibility of DNA for protein binding and enzymatic processing (Watson et al., 2008) (Figure 1A). The epigenome is established and maintained by the site-specific recruitment of chromatin-modifying enzymes and their cofactors. Identifying the *cis* elements that regulate transcription initiation and epigenomic modification is critical for understanding the regulatory mechanisms that control gene expression patterns.

Machine learning is used to predict the locations of TFBS and their TF binding specificities, other *cis*-regulatory elements and binding sites, larger DNA non-coding regions such as enhancers and promoters, as well as nucleosome binding landscapes and epigenetic states. The computational tasks for inferring TFBS from DNA sequence or modeling TFBS specificity based on TF activity measurements can be framed as binary/multiclass classification and regression problems, respectively. TFBS can be predicted from the genome *de novo* (Jayaram et al., 2016), or analyzed based on separate measurements (Kim et al., 2007; Visel et al., 2009; Ghandi et al., 2014) or massively parallel reporter assays using high-throughput quantitative sequencing technologies (HTS), giving peak calls for various regulatory (epigenetic and transcriptional) activities across tissues and isolated cell types (Project Consortium, 2012; Roadmap Epigenomics Consortium et al., 2015). These include: 1) ChIP-seq (Chromatin immunoprecipitation sequencing) (Johnson et al., 2007) and ChIP-nexus (addition of exonuclease digestion step) (He et al., 2015) to map TF binding sites and histone modification presence, 2) DNase-seq (DNase I hypersensitive sites sequencing) (Song and Crawford, 2010) and ATAC-seq (Assay for Transposase Accessible Chromatin with high-throughput sequencing) (Buenrostro et al., 2013) to measure DNA chromatin accessibility, which typically mark nucleosomes and TF-bound sites, and 3) other methods, such as PBMs (protein binding microarrays) (Berger et al., 2006), SELEX (Systematic evolution of ligands by exponential enrichment) (Blackwell and Weintraub, 1990) and BunDLE-seq [Binding to Designed Library, Extracting, and sequencing) (Levo et al., 2015) that can provide quantitative measurements of TF binding to thousands sequences within a single experiment (further details can be found in the following publication (Barshai et al., 2020)].

Common measures for evaluating the performance of ML classifiers, typically on unseen data, include: 1) precision and recall, 2) the area under the receiver operating characteristic curve (AUC) that measures the tradeoff between the true positive rate (recall) and false positive rate for different thresholds, as well as 3) the area under the precision recall curve (AUPRC) that measures the tradeoff between precision and recall for different thresholds [for technical details we refer the reader to a recent review (Jiao and Du, 2016)]. Regression models are frequently evaluated using a correlation coefficient or the coefficient of variation (*R* ^*2*^) (de Boer et al., 2020; Zrimec et al., 2020).

### Classical Machine Learning Relies on Engineered Features

**FIGURE 2:**
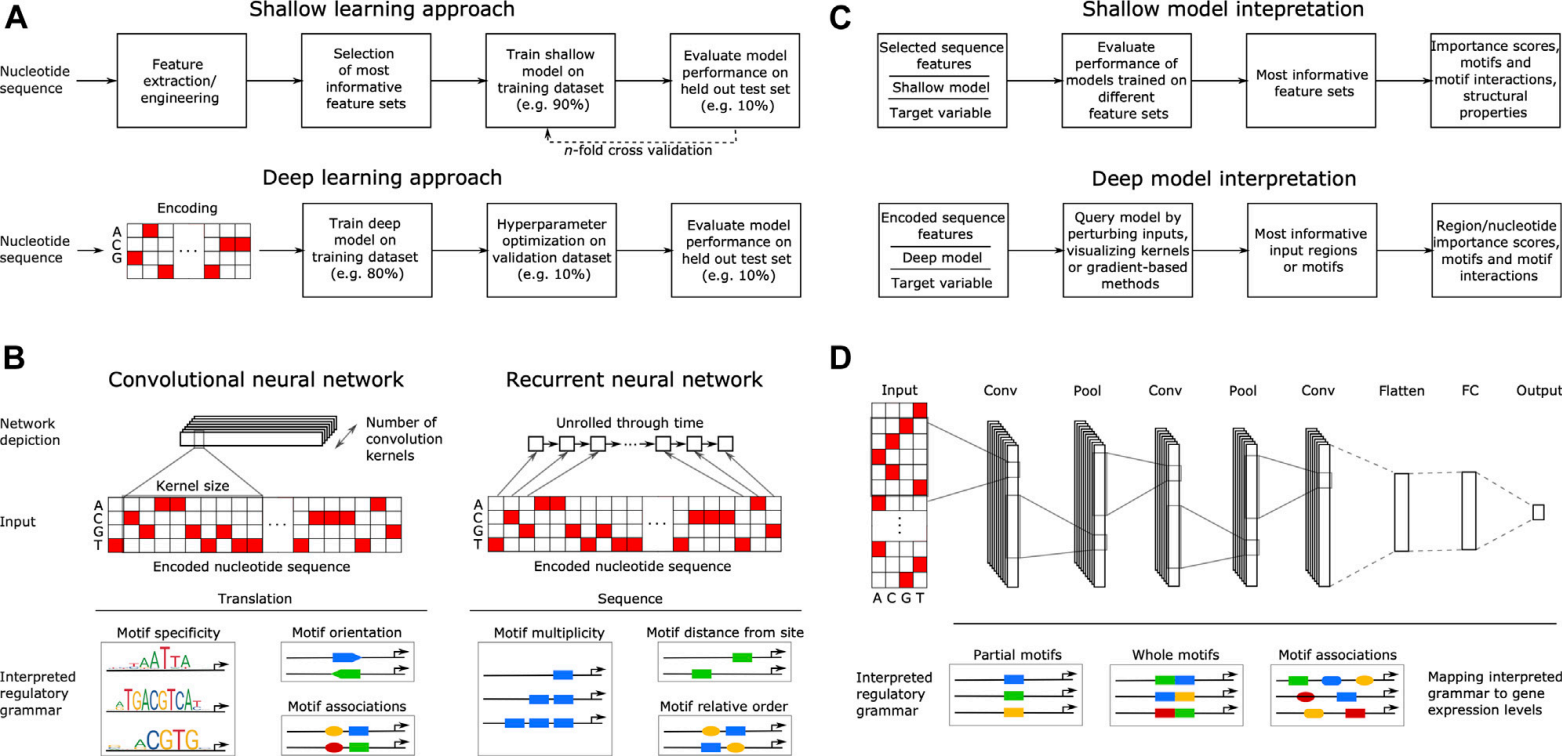
Principles of machine learning from nucleotide sequence. **(A)** Flowcharts of a typical supervised shallow modeling approach **(top)** and a typical supervised deep modeling approach **(bottom)**, depicting a one-hot encoding that equals k-mer embedding with *k* = 1. **(B)** Overview of convolutional (CNN) and recurrent neural networks (RNN) in interpreting DNA regulatory grammar. A CNN scans a set of motif detectors (kernels) of a specified size across an encoded input sequence, learning motif properties such as specificity, orientation and co-association. An RNN scans the encoded sequence one nucleotide at a time, learning sequential motif properties such as multiplicity, distance from e.g. transcription start site and the relative order of motifs. **(C)** Interpreting shallow models **(top)** by evaluating their performance when trained on different feature sets can yield feature importance scores, motifs and motif interactions, as well as compositional and structural properties. Similarly, interpreting the regulatory grammar learned by deep models **(bottom)**, by e.g. perturbing the input, visualizing kernels or using gradient-based methods, can yield feature importance scores spanning nucleotides up to whole regions, as well as motifs and motif interactions. **(D)** Example of a typical deep neural network (DNN) comprising three separate convolutional layers (Conv) connected *via* pooling layers (Pool) and a final fully connected network (FC) producing the output gene expression levels. Pool stages compute the maximum or average of each motif detector’s rectified response across the sequence, where maximizing helps to identify the presence of longer motifs and averaging helps to identify cumulative effects of short motifs. The DNN learns distributed motif representations in the initial Conv layers and motif associations that have a joint effect on predicting the target in the final Conv layer, representing DNA regulatory grammar that is mapped to gene expression levels.

The goal of supervised ML is to learn a response function *y* (target variable) from the set of features *x* (explanatory variables) present in the training dataset, where *y* describes some property related to gene expression, such as TF binding, ChIP-seq signal or mRNA abundance. With shallow learning, the DNA sequence that generally serves as the explanatory variable must be described with numerical features, such as position weight matrices (PWMs) (Stormo, 2000; Jayaram et al., 2016; Lu and Rogan, 2018), ungapped or gapped k-mer frequencies (Fletez-Brant et al., 2013; Ghandi et al., 2014; Zrimec et al., 2020), pseudo k-tuple nucleotide composition (Lin et al., 2014; Chen et al., 2015) or physicochemical and conformational (structural) properties (Rohs et al., 2009; Meysman et al., 2012; Zrimec, 2020a). Shallow methods thus require some features and methods that can describe or interpret the DNA regulatory motifs, and then use these features or motifs to build predictors. Due to their dependence on feature engineering, the shallow model training and evaluation methodology also commonly includes feature selection on all variables, retaining only the feature sets most informative for predicting the target variable. Afterward, ML models are trained on the engineered and selected feature subsets and finally, validation is performed on a held out portion of the data to assess the model performance (Ghandi et al., 2014; Zelezniak et al., 2018; Zrimec and Lapanje, 2018) (Figure 2A).

Comparison of 26 different approaches to model and learn a protein’s DNA-binding specificity based on PBMs for various mouse TFs (Weirauch et al., 2013) showed that, for most TFs examined, simple models based on mononucleotide PWMs can perform similarly to more complex models, falling short only in specific cases that represented less than 10% of the examined TFs. The best-performing motifs typically have relatively low information content, consistent with widespread degeneracy in eukaryotic TF sequence preferences. Out of multiple *de novo* motif discovery tools that can be used locally for creating PWMs from HTS data and for scanning them against DNA, FIMO (Grant et al., 2011) and MCast (Grant et al., 2016) were found to have the best performance in their respective classes of methods that predict individual TFBSs or identify clusters, respectively (Table 1) (Jayaram et al., 2016). In an approach termed “Catchitt” for predicting cell type-specific TFBS using ensemble classifiers (Keilwagen et al., 2019), standard PWM motifs from databases were expanded with motifs learned by *de novo* motif discovery from ChIP-seq and DNase-seq data using sparse local inhomogeneous mixture (Slim) models (Keilwagen and Grau, 2015), which capture short to mid-range intra-motif dependencies. Catchitt earned a shared first rank in the 2017 ENCODE-DREAM *in vivo* TFBS prediction challenge, achieving a median AUPRC of 0.41 on test data. Despite the success of PWM-based methods, ML approaches have been shown to achieve similar or even better results. For instance, the method “QBiC-Pred” was developed to quantitatively predict TF binding changes due to sequence variants (Martin et al., 2019), using ordinary least squares (OLS) regression and HTS data containing single nucleotide variants (SNVs). The OLS models of TF binding specificity were accurate in predicting mutational effects on TF binding *in vitro* and *in vivo* (*R* ^*2*^ up to 0.95), outperforming widely used PWM models as well as recently developed DNNs (Alipanahi et al., 2015) on the tested data. The problem with any ML approach using k-mers as features is that it becomes susceptible to noisy training k-mer frequencies once *k* becomes large. This was solved with methods for robust estimation of k-mer frequencies based on alternative feature sets, where gapped k-mers were introduced as a followup to the initial k-mer method “kmer-SVM” (Lee et al., 2011). The new classifier termed “gkm-SVM” predicted functional genomic regulatory elements with significantly improved accuracy compared to the original kmer-SVM, increasing the precision by up to 2-fold and achieving an AUC of 0.97 for TFBS prediction, compared to  0.91 with kmer-SVM (Ghandi et al., 2014). In this case however, the PWM-based classifier still outperformed both methods (AUC = 0.98).

**TABLE 1:** Overview of studies modeling protein-DNA interactions that govern the initiation of gene expression from nucleotide sequence properties. Highest achieved or average scores are reported, on test sets where applicable, and include precision (*prec*) and recall (*rec*), area under the receiver operating characteristic curve (AUC), area under the precision recall curve (AUPRC), the coefficient of variation (*R* ^*2*^), Pearson’s correlation coefficient (*r*), Spearman’s correlation coefficient (*ρ*) and Matthews correlation coefficient (MCC).

Ref.	Strategy	Target var.	Explan. vars.	Method	Score	Organism
(Jayaram et al., 2016)	Shallow	TFBS prediction	PWMs	PWM alignment algorithms	*prec* = 0.73, *rec*. = 0.82	Human
(Keilwagen et al., 2019)	Shallow	TFBS prediction	DNA motif and chromatin-based features	Classifier ensembles	AUPRC = 0.81	Human
(Ghandi et al., 2014)	Shallow	TFBS prediction	PWMs, gapped k-mers	SVM classification	AUC = 0.98	Human
(Levo et al., 2015)	Shallow	TF binding specificity	k-mers, DNA structural variables	L1-regularized LR	*R* ^*2*^ = 0.90	Yeast
(Yang et al., 2017)	Shallow	TF binding specificity	k-mers, DNA structural variables	L2‐regularized multiple LR	*R* ^*2*^ = 0.90	Human
(Martin et al., 2019)	Shallow	TF binding specificity	k-mers	OLS regression	*R* ^*2*^ = 0.95	Human
(Lin et al., 2014)	Shallow	σ54 promoter prediction	Pseudo k-tuple nucleotide composition	SVM classification	MCC = 0.88	*E. coli*
(He et al., 2018)	Shallow	σ70 promoter prediction	Trinucleotide-based features	SVM classification	MCC = 0.92	*E. coli*
(Benveniste et al., 2014)	Shallow	Histone modifications	k-mers, TF CHIP-seq data	LR classification	AUC = 0.95	Human
(Whitaker et al., 2015)	Shallow	Histone modifications, DNA methylation	DNA motifs	RF classification	AUC = 0.96	Human
(Lee et al., 2015)	Shallow	DNA chromatin accessibility	PWMs, gapped k-mers	SVM classification	AUC = 0.75	Human
(Trabelsi et al., 2019)	Deep	TFBS prediction	k-mers	CNN + biLSTM classification	AUC = 0.93	Human
(Zeng et al., 2016)	Deep	TFBS prediction	DNA sequence	CNN classification	AUC = 0.88	Human
(Kelley, 2020)	Deep	TFBS prediction	DNA sequence	CNN classification	AUC = 0.82	Human, mouse
(Chen et al., 2021)	Deep	TFBS prediction	DNA sequence	CNN + biLSTM + attention classification	AUC = 0.99	Human
(Alipanahi et al., 2015)	Deep	TF binding specificity	DNA sequence	CNN classification	AUC = 0.90	Human
(Wang et al., 2018)	Deep	TF binding specificity	DNA sequence	CNN regression	*ρ* = 0.81	Human
(Avsec et al., 2021)	Deep	TF binding specificity	DNA sequence	CNN regression	*ρ* = 0.62	Human
(Van Brempt et al., 2020)	Deep	Transcription initiation frequency	DNA sequence	CNN ordinal regression	*R* ^*2*^ = 0.88	*E. coli*
(Zhou and Troyanskaya, 2015)	Deep	Multitask chromatin profiling data	DNA sequence	CNN classification	AUC = 0.96	Human
(Quang and Xie, 2016)	Deep	Multitask chromatin profiling data	DNA sequence	CNN + biLSTM classification	AUC = 0.97	Human
(Park et al., 2020)	Deep	Multitask chromatin profiling data	DNA sequence	CNN + biLSTM + attention classification	AUC = 0.95	Human
(Singh et al., 2016)	Deep	Histone modifications	DNA sequence	CNN classification	AUC = 0.80	Human
(Singh et al., 2017)	Deep	Histone modifications	DNA sequence	LSTM + attention classification	AUC = 0.81	Human
(Kelley et al., 2016)	Deep	DNA chromatin accessibility	DNA sequence	CNN classification	AUC = 0.90	Human
(Kelley et al., 2018)	Deep	DNA chromatin accessibility	DNA sequence	CNN regression	*r* = 0.86	Human
(Angus and Eyuboglu, 2018)	Deep	DNA chromatin accessibility	DNA sequence	CNN + attention regression	*ρ* = 0.59	Human
(Angermueller et al., 2017)	Deep	DNA methylation	DNA sequence and features	CNN classification	AUC = 0.83	Human
(Tian et al., 2019)	Deep	DNA methylation	DNA sequence	CNN regression	AUC = 0.97	Human

In the case of epigenetic states that underlie DNA accessibility, it was shown that histone modifications can be predicted with remarkable accuracy from TF-binding profiles using LR classifiers (avg. AUC ∼0.86 to 0.95 on different DNA regions in H1 cells), recapitulating known interactions between TFs and chromatin-modifying enzymes (Benveniste et al., 2014). This demonstrated that associations between gene expression and histone modifications do not necessarily imply a direct regulatory role for these modifications, but can be explained equally well as an indirect effect of interactions between TFs and chromatin-modifying enzymes. Similarly, a pipeline termed “Epigram” (Whitaker et al., 2015) was developed to predict histone modification and DNA methylation patterns from DNA motifs. The authors also cataloged novel *cis* elements by *de novo* motif finding, showing that numerous motifs that have location preference and represented interactions with the site-specific DNA-binding factors that establish and maintain epigenomic modifications. Using their method gkm-SVM (Ghandi et al., 2014) to encode cell type–specific regulatory sequence vocabularies, Lee and colleagues (Lee et al., 2015) devised a sequence-based computational method to predict the effect of regulatory variation. The effect of sequence variants was quantified by the induced change in the gkm-SVM score, “deltaSVM,” which accurately predicted the impact of SNVs on DNase I hypersensitivity in their native genomes and could identify risk-conferring functional variants in validated data including autoimmune diseases, demonstrating the usefulness of this approach.

Apart from the base DNA sequence properties, structural properties have been found to improve model performance in certain cases, such as when predicting: 1) TFBS and their specificities (Abe et al., 2015; Tsai et al., 2015; Mathelier et al., 2016; Yang et al., 2017), 2) promoters and TSS sites (Meysman et al., 2012; Bansal et al., 2014; Kumar and Bansal, 2017), and 3) σ factor binding sites (Zrimec, 2020a). These properties are directly related to protein-DNA recognition and binding (Rohs et al., 2009; Bishop et al., 2011; Zrimec, 2020b) and include DNA shape (Mathelier et al., 2016), thermodynamic stability (SantaLucia, 1998) and propensity for duplex destabilization (Zrimec and Lapanje, 2015), as well as flexibility and curvature related properties (Brukner et al., 1995; Geggier and Vologodskii, 2010). For instance, the dependence of TF binding specificity on the TFBS core and flanking sequence was studied using LR and BunDLE-seq data on thousands of designed sequences with single or multiple Gcn4 or Gal4 binding sites (Levo et al., 2015). By supplanting k-mer frequencies at each position with DNA structural properties, 15 bp flanking sequences (15 bp) of core binding sites were shown to affect the binding of TFs, as models based on combined core and flanking regions explained the highest amount of variance in the measurements (*R* ^*2*^ up to 0.9 for Gal4). The contribution of DNA shape readout and its importance in core motif‐flanking regions was further demonstrated using LR and HT‐SELEX data across a diverse set of 215 mammalian TFs from 27 families (Yang et al., 2017), as regression models that used k-mer and shape features generally outperformed k-mer models by ∼10% (*R* ^*2*^ up to 0.90). Using feature selection techniques, positions in the TFBSs could be pinpointed where DNA shape readout is most likely to occur, and accordingly, novel DNA shape logos were proposed to visualize the DNA shape preferences of TFs. Similarly, SVM regression models of TF binding specificity based on PBM data for 68 mammalian TFs showed that shape-augmented models compared favorably to sequence-based models (Zhou et al., 2015), as DNA shape features reduced the dimensionality of the feature space. The authors from Rohs lab also provide an updated database of TFBS shape logos in 2020 (Chiu et al., 2020). Moreover, derivatives of DNA structural properties, such as pseudo k-tuple nucleotide compositions (Lin et al., 2014) and trinucleotide features including position-specific propensity and electron-ion potential (He et al., 2018), were applied to the problem of predicting bacterial σ54 and σ70 promoters in *E. coli*, which transcribe carbon and nitrogen-related genes or regulate the transcription of most genes, respectively. The respective ML classifiers termed “iPro54-PseKNC” (Lin et al., 2014) and “70ProPred” (He et al., 2018) could accurately distinguish the specific promoters from negative examples (AUC = 0.98 and 0.99, respectively).

### Deep Neural Networks can Learn Regulatory Grammar Automatically

In contrast to shallow architectures that are limited in their applications even when large datasets are available, deep architectures are abstracted by multiple hidden layers between *x* and *y*. Each layer learns a new representation of the data before passing it on to the successive layers, finding hidden data structures to make accurate predictions (Mhaskar et al., 2017). The most common DNN architectures in genomics include convolutional neural networks (CNNs) and recurrent neural networks (RNNs), such as bidirectional long short-term memory (biLSTM) networks. CNNs are regularized fully connected networks that progressively scan a DNA molecule within a receptive field, where they learn to recognize the occurrence of DNA motifs (e.g. specificity, orientation and co-association) (Eraslan et al., 2019a) (Figure 2B). Despite the capability of RNNs to learn sequential information (e.g. multiplicity, relative order), they are computationally expensive to train and certain improvements to CNNs, such as dilation (Yu and Koltun, 2015) and self-attention (Wang et al., 2017; Bello et al., 2019; Repecka et al., 2021), enable them to outperform RNNs (Gupta and Rush, 2017; Strubell et al., 2017; Trabelsi et al., 2019). Dilated convolution uses kernels with gaps to allow each kernel to capture information across a larger stretch of the input sequence, without incurring the increased cost of using RNNs (Gupta and Rush, 2017; Strubell et al., 2017). Similarly, self-attention is a special case of attention mechanism that allows kernels to focus on specific parts of the input when producing the output, allowing positions across the entire input sequence to interact and contribute to the result with different attention weights (Vaswani et al., 2017).

Deep learning does not require feature engineering or selection, since this is an inherent feature of the DNN learning process (Webb, 2018). However, it does require representing the categorical nucleotide sequence data numerically using an encoding scheme, such as one-hot, which transforms the sequence into a binary matrix with columns corresponding to each category. DNNs have thus been applied mostly on one-hot encoded nucleotide sequences as input (Eraslan et al., 2019a; Alipanahi et al., 2015), with recent reports showing that the use of k-mer embedding to represent the input sequences can improve model performance compared to one-hot encoding (itself a special case of k-mer embedding where *k* = 1) (Trabelsi et al., 2019). These inputs are well suited for comprehending the base DNA motif information as well as higher order interactions that describe the DNA regulatory grammar of gene expression (Eraslan et al., 2019a; Zrimec et al., 2020). Thus, DNNs achieve high predictive accuracies often surpassing those of models based on engineered features and, in our experience, using structural DNA properties does not lead to improved predictive performance with DNNs (Zrimec et al., 2020). Due to the large amount of model *hyper*parameters, such as network structure (e.g. number and size of kernels, Figure 2B) and training algorithm (e.g. learning rate), a special step termed hyperparameter optimization (Bergstra et al., 2015) is required for finding the best combinations of these hyperparameters and is an integral part of DNN training. To train DNNs, the data is typically split into training, validation, and testing datasets, where: 1) the model is trained on the training set by minimizing a loss function commonly MSE for regression and cross entropy for classification (Géron, 2019), 2) hyperparameter tuning is performed on the validation set and the best performing model on the validation set is chosen, and 3) the performance of the final model is evaluated on the testing set, also verifying if it overfits the data (Eraslan et al., 2019a; Zrimec et al., 2020) (Figure 2A). With DNN testing, cross-validation is rarely performed due to the large dataset sizes and issues with algorithmic efficiency. Commonly, 10% test splits are used for testing the models trained on 80% of the data, whereas another 10% of the training data is used for the internal validation of hyperparameter selection (Géron, 2019). For further technical details we refer the reader to excellent recent reviews (Eraslan et al., 2019a; Barshai et al., 2020).

Deep methods are frequently trained on HTS peak profiles, either converted to binary scores or left continuous as a regression problem, and the underlying TFBS and specificities are interpreted by the network itself. The first such method to showcase the efficiency of DNNs for analysis of TF binding specificities was DeepBind (Alipanahi et al., 2015), where a single CNN layer was trained on sequence specificities of DNA and RNA-binding proteins as measured by several types of HTS assays (including PBM, HT-SELEX, and ChIP-seq), in a combined 12 terabases of mouse and human data. DeepBind captured binding specificities from raw sequence data by jointly discovering new motifs of hundreds of TFs along with the rules for combining them into a predictive binding score. The resulting DeepBind models could then be used to identify binding sites in test sequences and to score the effects of novel mutations, uncovering the regulatory role of disease-associated genetic variants that can affect TF binding and gene expression. Importantly, the method outperformed 14 other methods (Weirauch et al., 2013) and achieved the highest score when applied to the *in vivo* ChIP-seq data (avg. AUC = 0.90), suggesting that it can generalize from HT-SELEX (Jolma et al., 2013) to other data acquisition technologies despite being based on a general-purpose ML framework.

The basic approach of DeepBind was further explored and expanded upon in subsequent studies with different network layers. For instance, Zeng and co. (Zeng et al., 2016). performed a systematic exploration of CNN architectures for predicting DNA sequence binding using a similarly large set of TF data. To control potentially confounding effects, like positional or motif strength bias, they chose to explore two specific classification tasks of motif discovery (bound vs. dinucleotide shuffles per TF and cell type) and motif occupancy (bound vs. non-bound). In both tasks, classification performance increased with the number of convolution kernels (AUC up to 0.88), and the use of local pooling or additional layers had little effect on the performance. CNN architectures that took advantage of these insights exceeded the classification performance of DeepBind, emphasizing the need to use sufficient kernels to capture motif variants. With deepRAM, a tool providing an implementation of a wide selection of architectures (Trabelsi et al., 2019), it was shown that deeper, more complex architectures provide a clear advantage with sufficient training data, with hybrid CNN + RNN architectures outperforming other methods in terms of accuracy (AUC = 0.93 with 1xCNN + biLSTM). However, although RNNs improve model accuracy, this comes at the expense of a loss in the interpretability of the features learned by the model. Kelley (Kelley, 2020) developed a strategy to train deep CNNs simultaneously on human and mouse genomes, which improved gene expression prediction accuracy on held out and variant sequences. Applying mouse regulatory models to analyze human genetic variants associated with molecular phenotypes and disease improved model performance (AUROC increased from 0.80 to 0.82), showing that the thousands of available non-human transcriptional and epigenetic profiles can be leveraged for more effective investigation of how gene regulation affects human disease. Moreover, the performance of assessing the functional impact of non-coding variants (e.g. SNVs) was further improved with DeFine (Wang et al., 2018), a regression model based on large-scale TF ChIP-seq data and capable of accurately predicting real-valued TF binding intensities (Spearman’s *ρ* up to 0.81). Here, the predicted changes in the TF binding intensities between the altered sequence and the reference sequence reflected the degree of functional impact for the variant, and could accurately identify the causal functional variants from measured disease-associated variants. Similar networks have also been used in bacteria, where the online promoter design tool (ProD) (Van Brempt et al., 2020) is based on forward engineering of promoter transcription initiation frequency (TIF). By training a CNN with high-throughput DNA sequencing data from fluorescence-activated cell sorted promoter libraries of *E. coli* σ70 and *Bacillus subtilis* σB-, σF- and σW-dependent promoters, prediction models were capable of predicting both TIF and orthogonality of the σ-specific promoters, which facilitated development of tailored promoters, where predictions explained ∼88% of the variance of experimental observations.

With prediction of epigenetic states, the “DeepSEA” method (Zhou and Troyanskaya, 2015) was the first to utilize three CNN layers trained for multi-task predictions of large-scale chromatin-profiling data, including transcription factor (TF) binding, DNase I hypersensitivity sites (DHSs) and histone-mark profiles across multiple cell types. The method significantly outperformed gkm-SVM (avg. AUC of 0.96 vs. 0.90) and enabled high-performance sequence-based prediction of both DHSs (avg. AUC = 0.92) and histone modifications (avg. AUC = 0.86). In the “DanQ” model (Quang and Xie, 2016) trained on similar data as DeepSEA, a hybrid CNN + RNN architecture was used in order to enhance its perception of regulatory grammar, where the CNN captured regulatory motifs and the RNN captured long-term dependencies between the motifs. The model achieved improved performance compared to DeepSEA (avg. AUC = 0.97) as well as compared to a LR baseline model, which despite its simplicity was an effective predictor (AUROC >0.70). Similarly, with histone modifications, the CNN “DeepChrome” (Singh et al., 2016) was shown to consistently outperform both SVM and RF classifiers (avg. AUC of 0.80 vs.  0.66 and 0.59, respectively). Kelley and co. (Kelley et al., 2016) introduced the open source package “Basset” that trains CNNs on a set of accessible genomic sites mapped in 164 cell types by DNase-seq, achieving improved predictive accuracy compared to previous methods, such as gkm-SVM (avg. AUC = 0.90 vs. 0.78), and good overlap of SNV predictions with previous observations. Furthermore, Kelley and co. (Kelley et al., 2018) developed another CNN, “Basenji,” to predict mammalian cell-type specific epigenetic and transcriptional profiles, where an unprecedented input sequence size of 131 kbp around TSS was used, spanning distal as well as proximal regulatory elements. Indeed, model predictions regarding the influence of SNVs on gene expression were shown to align well to known variants in human populations related to disease loci (avg. Pearson’s *r* = 0.86).

To map associations between DNA sequence patterns and methylation levels at CpG-site resolution, Angermuller and co. developed “DeepCpG” (Angermueller et al., 2017). The method was evaluated on single-cell methylation data across different cell types and HTS protocols, and yielded more accurate predictions than shallow methods, such as RF (avg. AUC = 0.83 vs. 0.80). The authors also showed that interpretation of the model parameters could provide insights into how sequence composition affects methylation variability. A more recent alternative approach termed “MRCNN” (Tian et al., 2019) outperformed DeepCpG (AUC up to 0.97), and *de novo* discovered motifs from the trained CNN kernels were shown to match known motifs.

Finally, by expanding DNN architectures with attention mechanisms to model complex dependencies among input signals, favourable results can be achieved compared to the non-attentive DNN counterparts. This was shown with multiple prediction tasks, including: 1) TFBS prediction, where “DeepGRN” (Chen et al., 2021) achieved higher unified scores in 6 of 13 targets than any of the top four methods in the 2016 ENCODE-DREAM challenge including Catchitt (Keilwagen et al., 2019), 2) histone modification, where “AttentiveChrome” (Singh et al., 2017) outperformed DeepChrome (Singh et al., 2016) in 50 out of 56 human cell types (avg. AUC of 0.81 vs. 0.80), 3) DNA chromatin accessibility, where the attention-based model (Angus and Eyuboglu, 2018) outperformed standard CNNs (*ρ* = 0.59 vs. 0.54) as well as dilated convolutions on specific experiments, and 4) multitask chromatin profiling data, where “TBiNet” (Park et al., 2020) outperformed DeepSea (Zhou and Troyanskaya, 2015) and DanQ (Quang and Xie, 2016) in the TF-DNA binding prediction task (avg. AUC of 0.95 vs. 0.90 and 0.93, respectively). This suggests that attention is an effective strategy to incorporate long-range sequence context into local predictions and particularly effective for gene-expression prediction.

### Interpreting Models to Retrieve the Learned Regulatory Grammar

With shallow models, the most informative feature sets are interpreted by evaluating the performance of models trained on different feature sets (Ghandi et al., 2014; Zrimec and Lapanje, 2018; de Boer et al., 2020) (Figure 2C). This can yield feature importance scores, motifs (k-mers or PWMs, depending on the provided input features, Figure 2A) and motif interactions (Ghandi et al., 2014; Keilwagen and Grau, 2015), as well as compositional and structural properties (Lin et al., 2014; Yang et al., 2017), all of which comprise a compendium of regulatory grammar, informative for understanding the regulation of gene expression. Due to the inherent capability of DNNs to learn predictive motif representations, rules for cooperative TF binding interactions (Avsec et al., 2021) and higher-order sequence features, such as secondary motifs and local sequence context (Zeng et al., 2016), as well as genotypic variation effects (Zhou and Troyanskaya, 2015), they represent a powerful approach to uncover the detailed *cis*-regulatory grammar of genomic sequences (Figure 2C) (Koo and Ploenzke, 2020a; He et al., 2020). This is achieved by interpreting the models using approaches that include: 1) CNN kernel visualization, where typically motifs in the initial layers are visualized, 2) input perturbation-based (sensitivity) analysis, which highlights the parts of a given input sequence that are most influential for the model prediction by occluding or mutating them (Alipanahi et al., 2015; Ancona et al., 2017), 3) gradient-based methods that estimate feature importance with iterative backward and forward propagations through the network (Shrikumar et al., 2017; Montavon et al., 2018; Shrikumar et al., 2018), yielding e.g. saliency maps (Simonyan et al., 2013) and 4) higher-order interactions among sequence elements, which can be assessed e.g. by using association rule analysis (Naulaerts et al., 2015; Zrimec et al., 2020), second-order perturbations (Koo et al., 2018), self-attention networks (Ullah and Ben-Hur, 2020) or by visualizing kernels in deeper layers (Maslova et al., 2020) [interested readers are referred to (Eraslan et al., 2019a; Koo and Ploenzke, 2020a)]. Moreover, attention mechanisms were recently shown to be more effective in discovering known TF-binding motifs compared to non-attentive DNNs (Park et al., 2020), as the learned attention weights correlate with informative inputs, such as DNase-Seq coverage and DNA motifs (Chen et al., 2021), and they can provide better interpretation than other established feature visualization methods, such as saliency maps (Lanchantin et al., 2016; Singh et al., 2017).

Since these are computational approaches, they extract statistical patterns that may not immediately reflect physical properties of the variables and should be treated as hypotheses that need to be further examined (Koo and Eddy, 2019). For instance, a method can point out certain motifs or associations that are important for the model in predicting the target, but how this reflects actual physicochemical interactions can be rather hard to interpret from the model alone. Nevertheless, this is an active area of research and new solutions are frequently developed (Lundberg and Lee, 2017; Chen and Capra, 2020; Koo and Ploenzke, 2020b), where rigorous testing as well as experimentally verifying predictions will highlight the most promising approaches (Ancona et al., 2017). On the other hand, an alternative trend that is arguably more appropriate than interpreting black box models is the development of inherently interpretable models (Rudin, 2019), where prior knowledge of gene expression can be built into the deep network structure itself (Ma et al., 2018; Tareen and Kinney, 2019; Liu et al., 2020). We refer interested readers to the excellent recent review by Azodi and co. (Azodi et al., 2020).

## Regulatory Mechanisms in Specific Coding and Non-Coding Regions

Both transcription and translation comprise multiple steps that include initiation, elongation and termination (Watson et al., 2008). Transcription of protein coding genes is controlled *via* the gene regulatory structure, comprised of coding and *cis*-regulatory regions that include promoters, untranslated regions (UTRs) and terminators, and generally proceeds in the direction from the upstream 5′ to downstream 3′ end (Figure 1B). Initiation is regulated by enhancers, promoters and 5′ UTRs, where the transcriptional machinery including RNA polymerase (RNAP) is guided to the correct sites on the DNA. In the elongation phase, mRNA is synthesized (transcribed) from the coding sequence, and this process terminates toward the 3′ UTR and terminator regions carrying termination signals. Afterward, the process of mRNA decay is triggered, which occurs in eukaryotes after the mRNA strand is matured by 5′ capping and 3′ poly(A) tail extension, and precursor mRNA (pre-mRNA) transcripts are processed by the spliceosome, removing introns (non-coding regions) and joining exons (coding regions) together (Watson et al., 2008; Wilkinson et al., 2020). The rates of mRNA synthesis and decay define the actual mRNA levels in the cell that are commonly measured with RNA-Seq (Wang et al., 2009). The DNA regions involved in mRNA synthesis carry multiple regulatory motifs, with codon usage in coding regions detailing which nucleotide triplets encoding an amino acid (AA) are used at each position, contributing to the base regulatory grammar of transcription (Plotkin and Kudla, 2011; Cheng et al., 2017). As described above, the general genomic architecture, defined by binding of histones in eukaryotes (Struhl and Segal, 2013) and nucleoid-associated proteins (NAPs) in prokaryotes (Dillon and Dorman, 2010), acts as a master regulator of transcription by controlling the accessibility of DNA to proteins (Curran et al., 2014; Morse et al., 2017).

Translation also proceeds in the direction from the 5′ to the 3′ end of an mRNA (Figure 1C) and, in bacteria, occurs simultaneously with transcription in the cytoplasm of the cell, whereas in eukaryotes transcription occurs in the nucleus and translation occurs in the cytoplasm (Watson et al., 2008). Prokaryotic mRNAs have a ribosome binding site (RBS) located in the 5′ UTR that aids recruitment of the translation machinery (Omotajo et al., 2015). In eukaryotes, mRNAs are modified at their 5′ and 3′ ends to facilitate translation by 5′ capping, which recruits the ribosome to the mRNA, and addition of a 3′ poly(A) tail, promoting higher translation by efficient recycling of ribosomes (Mayr, 2017). The key factors for initiation are ribosome recruitment to the mRNA and correct positioning over the start codon, where the presence of a Kozak sequence in the 5′ UTR also increases the efficiency of translation (Nakagawa et al., 2008; Hinnebusch et al., 2016). Elongation is mostly driven by codon usage, where ribosomes synthesize proteins by concatenating one AA per codon according to the genetic code (Saier, 2019). In the termination phase, release factors terminate translation in response to stop codons and the ribosomes are recycled.

### Open Reading Frame and Coding Region

Alternative splicing plays a crucial role for protein diversity in eukaryotic cells and produces several mRNA molecules from a single pre-mRNA molecule with ∼95% of human genes (Wilkinson et al., 2020). Conversely, in yeast, ∼6% of genes carry introns and very few alternative splice forms exist. RNA splicing requires a mandatory set of splicing signals including: 1) the splice donor site (5’ss) and splice acceptor site (3’ss) that define the exon/intron junction of each intron at the 5′ and 3′ ends, respectively, and are characterized by highly conserved dinucleotides (mainly GT and AG, respectively), and 2) the branch point site, a short and degenerate motif usually located between 18 and 44  bp upstream of 3’ss and as far as 400 bp upstream (Mercer et al., 2015). Alterations of these signals were found to be the most frequent cause of hereditary disease (Anna and Monika, 2018). Since 5’ss and 3’ss sequences are well characterized, reliable tools dedicated to splice site predictions have emerged, such as the logistic regression-based “SPiCE” (Leman et al., 2018), trained on 395 splice-site variants of 11 human genes, which achieved an accuracy of 95.6% and correctly predicted the impact on splicing for 98.8% of variants (Table 2). To predict the position of splice sites on long genomic sequences, “SpliceRover” (Zuallaert et al., 2018) and “SpliceFinder” (Wang et al., 2019) were developed using CNNs, both outperforming existing splice site prediction tools. SpliceRover achieved ∼10% improvement over an existing SVM-based model (Sonnenburg et al., 2007) (AUPRC = 0.61 vs. 0.54) and SpliceFinder compared favourably to both LSTM and SVM-based approaches (AUC of 0.98 vs. 0.95 and 0.93, respectively). A deeper, 32-layer CNN termed “SpliceAI” that accurately predicts splice junctions in pre-mRNAs was developed by Jaganathan and co. (Jaganathan et al., 2019), enabling precise prediction of noncoding genetic variants that cause cryptic splicing and outperforming shallow methods (AUPRC = 0.98 vs. 0.95). The study also found that splice-altering mutations are significantly enriched in patients with rare genetic disorders, causing an estimated 9–11% of pathogenic mutations. For identification of relevant branch points, the method “Branchpointer” (Signal et al., 2018) based on gradient boosting machines showed the best performance to detect the branch points upstream of constitutive and alternative 3’ss (accuracy of 99.48 and 65.84%, respectively). Alternatively, for variants occurring in a branch point area, the mixture-model based “BPP” (Zhang et al., 2017a) emerged as having the best performance to predict effects on mRNA splicing, with an accuracy of 89.17%. Interestingly, two deep learning methods based on bidirectional LSTMs, “LaBranchoR” (Paggi and Bejerano, 2018) and “RNABPS” (Nazari et al., 2019), both performed worse than the above shallow methods when assessed on large scale datasets (AUC of 0.71 and 0.81, respectively, vs. 0.82 with BPP using constitutive 3’ss) (Leman et al., 2020).

**TABLE 2:** Overview of studies modeling gene expression-related properties from separate regulatory or coding regions. Highest achieved or average scores are reported, on test sets where applicable, and include accuracy (*acc*), area under the receiver operating characteristic curve (AUC), area under the precision recall curve (AUPRC), the coefficient of variation (*R* ^*2*^) and Pearson's correlation coefficient (*r*).

Ref.	Strategy	Region	Target var.	Explan. vars.	Method	Score	Organism
(Leman et al., 2018)	Shallow	Coding	Splice site prediction	Sequence and PWM features	Logistic regression	*acc* = 0.96%	Human
(Signal et al., 2018)	Shallow	Coding	Branch point prediction	Sequence features	Gradient boosting classification	AUC = 0.94	Human
(Zhang et al., 2017a)	Shallow	Coding	Branch point prediction	Sequence features	Mixture models classification	AUC = 0.82	Human
(Trösemeier et al., 2019)	Shallow	Coding	Protein abundance	Codon usage features	COSEM mathematical model	*R* ^*2*^ = 0.45, 0.51, 0.37, respectively	*E. coli*, yeast, human
(Ferreira et al., 2020)	Shallow	Coding	Protein abundance	Codon usage	AdaBoost regression	*R* ^*2*^ = 0.95	Yeast
(Tunney et al., 2018)	Shallow	Coding	Ribosome density at each codon	Codon usage	NN regression	*r* = 0.57	Yeast
(Zuallaert et al., 2018)	Deep	Coding	Splice site prediction	DNA sequence	CNN classification	AUPRC = 0.61	Human, *A. thaliana*
(Wang et al., 2019)	Deep	Coding	Splice site prediction	DNA sequence	CNN classification	AUC = 0.98	Human
(Jaganathan et al., 2019)	Deep	Coding	Splice site prediction	DNA sequence	CNN classification	AUPRC = 0.98	Human
(Paggi and Bejerano, 2018)	Deep	Coding	Branch point prediction	DNA sequence	biLSTM classification	AUC = 0.71	Human
(Nazari et al., 2019)	Deep	Coding	Branch point prediction	DNA sequence	biLSTM + CNN classification	AUC = 0.81	Human
(Xu et al., 2017)	Deep	Coding	Alternative splicing prediction	Sequence and epigenetic features	Dense DNN classification	AUPRC = 0.89	Human
(Lee et al., 2020)	Deep	Coding	Alternative splicing prediction	Sequence and epigenetic features	RNN classification	AUPRC = 0.8	Human
(Zhang et al., 2019)	Deep	Coding	Alternative splicing prediction	RNA-seq data	Dense DNN + bayesian hypothesis testing	AUC = 0.87	Human
(Fu et al., 2020)	Deep	Coding	Protein abundance	DNA sequence	Multilayer biLSTM regression	*R* ^*2*^ = 0.52	*E. coli*
(Fujimoto et al., 2017)	Deep	Coding	Optimal codon usage	DNA sequence	biLSTM encoder-decoder	*acc* = 0.97	*E. coli*
(Yang et al., 2019)	Deep	Coding	Transcript abundance	DNA sequence	biLSTM transducer	*acc* = 0.67	*E. coli*, human
(Grossman et al., 2017)	Shallow	Enhancer	Transcript abundance	Motifs and pairwise motif interactions	L1-regularized LR	*R* ^*2*^ = 0.38 (natural), 0.52 (synthetic)	Human
(Lee et al., 2011)	Shallow	Enhancer	Enhancer prediction	k-mers	SVM classification	AUC = 0.93	Human
(Min et al., 2017)	Deep	Enhancer	Enhancer prediction	DNA sequence	CNN classification	AUPRC = 0.92	Human
(Cohn et al., 2018)	Deep	Enhancer	Enhancer prediction	DNA sequence	CNN classification	AUC = 0.92	17 mammalian species including human
(Niu et al., 2019)	Deep	Enhancer	Transcript abundance	DNA sequence	CNN regression	AUC = 0.92	Human
(Chen and Capra, 2020)	Deep	Enhancer	Multitask regulatory properties	DNA sequence	Deep residual NN classification	AUPRC = 0.98	Human
(Lubliner et al., 2015)	Shallow	Promoter	Core promoter activity via reporter fluorescence	k-mers	LR	*R* ^*2*^ = 0.72	Yeast
(Urtecho et al., 2019)	Shallow	Promoter	mRNA abundance	σ factor binding sites	NN regression	*R* ^*2*^ = 0.96	*E. coli*
(Einav and Phillips, 2019)	Shallow	Promoter	mRNA abundance	σ factor binding sites	Biophysical model	*R* ^*2*^ = 0.91	*E. coli*
(de Boer et al., 2020)	Shallow	Promoter	Protein abundance	TF binding and sequence features	L2-regularized multiple LR	*R* ^*2*^ = 89 (natural), 94 (synthetic)	Yeast
(Hossain et al., 2020)	Shallow	Promoter	mRNA abundance	TF binding and sequence features	L1-regularized multiple LR	*R* ^*2*^ = 0.49	*E. coli*, yeast
(Leiby et al., 2020)	Deep	Promoter	Transcription initiation rate	DNA sequence	CNN regression	*R* ^*2*^ = 0.90	*E. coli*
(Kotopka and Smolke, 2020)	Deep	Promoter	Protein abundance	DNA sequence	CNN regression	*R* ^2^ = 0.79	Yeast
(Dvir et al., 2013)	Shallow	5′ UTR	Protein levels	DNA sequence features + k-mers	LR	*R* ^*2*^ = 0.52	Yeast
(Bonde et al., 2016)	Shallow	5′ UTR	Protein abundance	RBS features	RF regression	*R* ^*2*^ = 0.89	*E. coli*
(Salis et al., 2009; Salis, 2011)	Shallow	5′ UTR	Protein abundance	RBS features	Thermodynamic model, LR	*R* ^*2*^ = 0.54 (natural), 0.84 (synthetic)	*E. coli*
(Espah Borujeni et al., 2017)	Shallow	5′ UTR	Translation initiation rate	N-terminal mRNA structures	Biophysical model, LR	*R* ^*2*^ = 0.78	*E. coli*
(Ding et al., 2018)	Shallow	5′ UTR	Protein abundance	DNA sequence activity relationships	Partial least-squares (PLS) regression	*R* ^*2*^ = 0.60 (natural), 0.71 (synthetic)	Yeast
(Decoene et al., 2018)	Shallow	5′ UTR	Translation initiation rate	DNA sequence features	PLS regression	*R* ^*2*^ = 0.73	Yeast
(Cuperus et al., 2017)	Deep	5′ UTR	Protein abundance	DNA sequence	CNN regression	*R* ^*2*^ = 0.62	Yeast
(Sample et al., 2019)	Deep	5′ UTR	Mean ribosome load	DNA sequence	CNN regression	*R* ^*2*^ = 0.82	Human
(Morse et al., 2017)	Shallow	3′ UTR, terminator	Protein abundance	Nucleosome occupancy score	Weighted LR	*R* ^*2*^ = 0.84	Yeast
(Cambray et al., 2013)	Shallow	Terminator	Termination efficiency	DNA sequence features (12)	Multiple LR	*r* = 0.9	*E. coli*
(Vogel et al., 2010)	Shallow	3′ UTR, terminator	mRNA abundance	k-mers	L1-regularized logistic regression	*r* = 0.41	Yeast
(Bogard et al., 2019)	Deep	3′ UTR	Alternative polyadenylation signals	DNA sequence	CNN regression	*R* ^*2*^ = 0.88	Human

Further deep learning studies on alternative splicing prediction have shown that a comprehensive splicing code should include not only genomic sequence features but also epigenetic properties. For instance, 16 histone modifications were used with a multi-label DNN for human embryonic stem cell differentiation in an approach termed “DeepCode” (Xu et al., 2017), achieving an AUPRC up to 0.89. Lee and co. (Lee et al., 2020) built an interpretable RNN that mimics the physical layout of splicing regulation, where the chromatin context progressively changes as the RNAP moves along the guide DNA, achieving an AUPRC of over 0.8 and showing that adjacent epigenetic signals carry useful information in addition to the actual nucleotide sequence of the guide DNA strand. Finally, to enable the characterization of differential alternative splicing between biological samples based on RNA-seq datasets even with modest coverage, the approach DARTS (Zhang et al., 2019) was developed based on a DNN and a Bayesian statistical framework used to determine the statistical significance of differential splicing events in RNA-seq data across biological conditions.

The genetic code is degenerate as most AAs are coded by multiple codons, and these codons would appear in equal frequencies if use of specific codons would not amount to any change in cellular fitness. However, the unequal use of codons that decode the same AA, termed codon usage bias (CUB), cannot be explained by mutation bias alone and is generally believed to arise from selection for improved translational efficiency (Plotkin and Kudla, 2011). Due to variations in transfer RNA (tRNA) abundances, favoring the usage of codons that correspond to more abundant tRNA can lead to faster translation. Such codons are preferred or “optimal” for translation speed up (termed codon optimality) (Hershberg and Petrov, 2008). This is supported by multiple findings in both prokaryotes and eukaryotes, showing that CUB correlates with translation efficiency (protein numbers per mRNA) (Tuller et al., 2010), certain protein structural motifs and tRNA levels (Hanson and Coller, 2018), and affects mRNA translation initiation rates and elongation rates. Furthermore, CUB indices of genes, such as the codon adaptation index (CAI) (Sharp and Li, 1987; Carbone et al., 2003), tend to correlate with the genes’ expression (Ghaemmaghami et al., 2003). The role of the coding region extends beyond codon usage, however. mRNA structure was found to regulate translation (Yu et al., 2019) and mRNA hairpins can obstruct translation and override the effect of codon usage bias on translation (Cambray et al., 2018).

The strong association of mRNA levels with protein expression in a variety of organisms (Schwanhäusser et al., 2011; Csárdi et al., 2015; Liu et al., 2016) indicates a more complex background process. The selection pressure for increased protein expression can manifest in changes of DNA that optimize both translation and transcription, improving protein expression and mRNA levels, respectively. Multiple lines of recent evidence corroborate this dual role of synonymous codon changes in transcription and translation, suggesting that selection is shaping codon usage not only to optimize translational efficiency, but in response to conditions imposed by the transcription machinery as well as the physical properties of mRNA (Zhou et al., 2016; Zhou et al., 2018b). For instance, in fungi, codon optimization was found to increase mRNA and protein levels in a promoter-independent manner (Zhou et al., 2016), with CUB shown to be predictive of mRNA and protein levels, affect mRNA stability (Presnyak et al., 2015) and toxicity (Mittal et al., 2018), coevolve with transcription termination (Zhou et al., 2018b) as well as be influenced by mRNA local secondary structure (Trotta, 2013). Similarly, in *E. coli*, CUB was found to affect mRNA stability by defining mRNA folding at the ribosomal site (Kudla et al., 2009).

Multiple modeling studies have been performed to analyze the causes and effects of CUB as well as to find ways to optimize codon usage in order to boost gene expression levels. Codon optimization is a mature field with tools readily available on most biotechnology and DNA synthesis companies’ websites (e.g. www.thermofisher.com, www.genewiz.com, www.twistbioscience.com) as well as in standalone solutions (Puigbò et al., 2007; Gould et al., 2014; Rehbein et al., 2019). Most existing optimization strategies are based on biological indices, such as CAI (Sharp and Li, 1987; Puigbò et al., 2007), and use the host’s preferred codons to replace less frequently occurring ones, while also adjusting the new sequences to match the natural codon distribution in order to preserve the slow translation regions that are important for protein folding (Richardson et al., 2006; Angov et al., 2008; Hershberg and Petrov, 2009; Gaspar et al., 2012). Standard codon usage metrics were shown to be highly predictive of protein abundance. For instance, an AdaBoost model trained on a number of codon usage metrics in *S. cerevisiae* genes coding for high-abundance proteins (top 10%) and low-abundance proteins (lowest 10%) was highly predictive of these extremes of protein abundance (*R* ^2^ = 0.95) (Ferreira et al., 2020).

However, while explicitly modeling existing frequency-based indices has helped to engineer high-yield proteins, it is unclear what other biological features (e.g. RNA secondary structure) should be considered during codon selection for protein synthesis maximization. To address this issue, inspired by natural language processing, deep learning was recently also applied to model CUB. Fujimoto and co. (Fujimoto et al., 2017) showed that their biLSTM-based deep language model that “translates” from DNA to optimal codon sequences, is more robust than existing frequency-based methods due to its reliance on contextual information and long-range dependencies. Similarly, a biLSTM-Transducer model of codon distribution in highly expressed bacterial and human transcripts was able to predict the next codon in a genetic sequence with improved accuracy and lower perplexity on a held out set of transcripts, outperforming previous state-of-the-art frequency-based approaches (accuracy of 0.67 vs. 0.64) (Yang et al., 2019). Another deep learning-based codon optimization approach introduced the concept of *codon boxes*, enabling DNA sequences to be transformed into codon box sequences, while ignoring the order of bases, and thus converting the problem of codon optimization to sequence annotation of corresponding AAs with codon boxes (Fu et al., 2020). Sequences optimized by these biLSTM codon optimization models with ones optimized by Genewiz and ThermoFisher were compared using protein expression experiments in *E. coli*, demonstrating that the method is efficient and competitive.

Alternatively, an algorithmic approach to replacing codons by the target organism’s preferred codons was developed by Trösemeier and co. (Trösemeier et al., 2019), termed “COSEM,” which simulates ribosome dynamics during mRNA translation and informs about protein synthesis rates per mRNA in an organism and context-dependent way. Protein synthesis rates from COSEM were integrated with further relevant covariates such as translation accuracy into a protein expression score that was used for codon optimization, with further algorithmic fine-tuning implemented in their software “OCTOPOS.” The protein expression score produced competitive predictions on proteomic data from prokaryotic and eukaryotic expression systems and was shown to be superior to standard methods, achieving 3-fold increases in protein yield compared to wildtype and commercially optimized sequences (Trösemeier et al., 2019). Moreover, since ribosomes do not move uniformly along mRNAs, Tunney and co. (Tunney et al., 2018) modeled the variation in translation elongation by using a shallow NN to predict the ribosome density at each codon as a function of its sequence neighborhood. This enabled them to study sequence features affecting translation elongation and to design synonymous variants of a protein coding sequence in budding yeast that closely tracked the predicted translation speeds across their full range *in vivo*, demonstrating that control of translation elongation alone is sufficient to produce large quantitative differences in protein output.

### Enhancer and Promoter

Transcriptional enhancers are located upstream of the transcription start site (TSS) and regulate spatiotemporal tissue-specific gene expression patterns over long genomic distances, which is achieved through the binding of TFs to cognate motifs (Shlyueva et al., 2014). They can typically be found farther away from the TSS with increasing genomic complexity of the organism (Mora et al., 2016; Clément et al., 2018; Zicola et al., 2019), as far as a million bps in mammals (Pennacchio et al., 2013). Enhancer function and TF binding are influenced by various features, such as the chromatin state of the genomic locus, binding site affinities, activity of bound TFs as well as interactions among TFs (Shlyueva et al., 2014; Chen and Capra, 2020). The nature of how TF interactions influence enhancer function was explored in a recent systematic analysis using *in vivo* binding assays with 32,115 natural and synthetic enhancers (Grossman et al., 2017). The activity of enhancers that contain motifs for PPARγ, a TF that serves as a key regulator of adipogenesis, were shown to depend on varying contributions from dozens of TFs in their immediate vicinity. Importantly, different pairs of motifs followed different interaction rules, including subadditive, additive, and superadditive interactions among specific classes of TFs, with both spatially constrained and flexible grammars.

One of the key ML tasks shedding new light on DNA features affecting enhancer function is identification of enhancer regions in genomic sequences. For instance, a k-mer based SVM framework was able to accurately identify specific types of enhancers (EP300-bound) using only genomic sequence features (Lee et al., 2011), outperforming PWM-based classifiers (AUC = 0.93 vs. 0.87). The predictive sequence features identified by the SVM classifier revealed both enriched and depleted DNA sequence elements in the enhancers, many of which were found to play a role in specifying tissue-specific or developmental-stage-specific enhancer activity, and others that operate in a general or tissue-independent manner. The first deep learning approach to facilitate the identification of enhancers, termed “DeepEnhancer” (Min et al., 2017), relied purely on DNA sequences to predict enhancers using CNNs and transfer learning to fine-tune the model on cell line-specific enhancers. The method was superior to gkm-SVM by ∼7% in both AUC and AUPRC scores, and visualizing CNN kernels as sequence logos identified motifs similar to those in the JASPAR database (Khan et al., 2018). Similarly, Cohn and co. (Cohn et al., 2018). trained deep CNNs to identify enhancer sequences in 17 mammalian species using simulated sequences, *in vivo* binding data of single TFs and genome-wide chromatin maps of active enhancers. High classification accuracy was obtained by combining two training strategies that identified both short (1–4 bp) low-complexity motifs and TFBS motifs unique to enhancers. The performance improved when combining positive data from all species together, demonstrating how transfer of learned parameters between networks trained on different species can improve the overall performance and supporting the existence of a shared mammalian regulatory architecture. Although identification of enhancer locations across the whole genome is necessary, it can be more important to predict in which specific tissue types they will be activated and functional. The existing DNNs, though achieving great successes in the former, cannot be directly employed in tissue-specific enhancer predictions because a specific cell or tissue type only has a limited number of available enhancer samples for training. To solve this problem, Niu and co. (Niu et al., 2019) employed a transfer learning strategy, where models trained for general enhancer predictions were retrained on tissue-specific enhancer data and achieved a significantly higher performance (geometric mean of precision and recall, GM = 0.81 vs. 0.70), also surpassing gkm-SVM (GM = 0.53). Interestingly, a very small amount of retraining epochs (∼20) were required to complete the retraining process, giving insight into the tissue-specific regulatory rewiring and suggesting that tissue specific responses are mediated by precise changes on a small subset of binding features.

Promoters are adjacent regions directly upstream, as well as a short distance downstream, of the TSS typically spanning from 50 to a couple of 100 bp (Sharon et al., 2012; Redden and Alper, 2015). Besides TFBS and enhancers, they contain core promoters (Lubliner et al., 2015; Haberle and Stark, 2018) in eukaryotes and σ factor binding sites (Feklístov et al., 2014) in prokaryotes, to which the RNAP is recruited and where it acts to initiate transcription. The core promoter contains several motifs with fixed positioning relative to the TSS (Haberle and Stark, 2018), including: 1) the TATA-box motif (consensus 5′-TATAWAW-3′), located ∼30  bp upstream of TSS and conserved from yeast to humans but found only in a minority of core promoters, 2) the initiator (Inr) motif, which directly overlaps the TSS and is more abundant than the TATA-box but not universal, with differing consensus sequence among organisms, 3) the downstream promoter element (DPE) that can accompany Inr in promoters that lack a TATA-box and is positioned downstream of the TSS, and 4) other motifs with defined positions relative to the TSS, including TFIIB recognition elements (BREs) and downstream core elements (DCEs) in humans (Watson et al., 2008; Haberle and Stark, 2018). A comprehensive study of yeast core promoter activity and TSS locations in thousands of native and designed sequences (Lubliner et al., 2015) showed that core promoter activity is highly correlated to that of the entire promoter and is in fact predictable from the sequence variation in core promoters (*R* ^*2*^ up to 0.72). Interestingly, orthologous core promoters across yeast species have conserved activities, with transcription initiation in highly active core promoters focused within a narrow region and location, orientation, and flanking bases critically affecting motif function. De Boer and co. (de Boer et al., 2020) recently transcended the limitations of using native and engineered sequences with insufficient scale, instead measuring the expression output of >100 million fully random synthetic promoter sequences in yeast. Using shallow ML they built interpretable models of transcriptional regulation that predicted 94 and 89% of the expression driven from independent test promoters and native yeast promoter fragments, respectively, with a deep model mentioned to have achieved 96%. These models allowed them to characterize each TF’s specificity, activity and interactions with chromatin, showing that expression level is influenced by weak regulatory interactions, which confound designed-sequence studies, further supporting that interactions between elements in regulatory regions play an important role in orchestrating gene expression. Moreover, based on promoter libraries comprising >1,000,000 constitutive and inducible promoters and using deep learning, Kotopka and Smolke (Kotopka and Smolke, 2020) developed accurate predictors of promoter activity (*R* ^*2*^ = 0.79) that were used for model-guided design of large, sequence-diverse promoter sets, confirmed to be highly active *in vivo*.

Prokaryotic promoters are marked by σ factor binding sites with five distinct motifs controlling transcription initiation rates by mediating RNAP recruitment: the −35, extended −10, −10, and discriminator motifs recognized by σ; and the UP element recognized by other RNAP domains (Browning and Busby, 2004; Feklístov et al., 2014). The −35 (consensus 5′-TTGACA-3′) and −10 motifs (consensus 5′-TATAAT-3′) are the most abundant, though the extended −10 motif can supplant −35 for initiation, both of which are recognized as dsDNA, with the remaining motifs recognized as ssDNA (Feklístov et al., 2014). By building and testing a library of 10,898 σ70 promoter variants consisting of combinations of −35, −10 and UP elements, spacers, and backgrounds in *E. coli* (Urtecho et al., 2019), the −35 and −10 sequence elements were shown to explain over 95% of the variance in promoter strength using a shallow NN. This was an improvement over using a simple log-linear statistical model, which explained ∼74% of the variance, likely due to capturing nonlinear interactions with the spacer, background, and UP elements. Based on the same data from Urtecho and co. (Urtecho et al., 2019), the central claim in energy matrix models of gene expression, stating that each promoter element contributes independently and additively to gene expression and contradicting experimental measurements, was tested using biophysical models (Einav and Phillips, 2019). A “multivalent” modeling framework incorporated the effect of avidity between the –35 and –10 RNAP binding sites and could successfully characterize the full suite of gene expression data (*R* ^*2*^ = 0.91), suggesting that avidity represents a key physical principle governing RNAP-promoter interaction, with overly tight binding inhibiting gene expression. Another use of the data by Urtecho and co. (Urtecho et al., 2019) was with deep learning, where CNN models were trained to predict a promoter’s transcription initiation rate directly from its DNA sequence without requiring expert-labeled sequence elements (Leiby et al., 2020). The model performed comparably to the above shallow models (*R* ^*2*^ = 0.90) and corroborated the consensus −35, −10 and discriminator motifs as key contributors to σ70 promoter strength. Similarly, using a “Nonrepetitive Parts Calculator” to rapidly generate and experimentally characterize thousands of bacterial promoters with transcription rates that varied across an almost 1e6-fold range, a ML model was built to explain how specific interactions controlled the promoters’ transcription rates, supporting that the number of −35 and −10 motif hexamer mismatches is a potent sequence determinant (Hossain et al., 2020).

### 5′ Untranslated Region

The key known sequence elements affecting gene expression in 5′ UTRs are the RBS, known as the Shine-Dalgarno sequence, in prokaryotes (Omotajo et al., 2015) and the Kozak sequence in eukaryotes (Nakagawa et al., 2008). The Shine-Dalgarno sequence is a ∼6 bp highly conserved sequence (consensus 5′-AGGAGG-3′) (Shine and Dalgarno, 1975) located 3–9 bp from the start codon, which aids recruitment of the ribosome to the mRNA and has a strong effect on the translation initiation rate, thus being highly predictive of expression (Bonde et al., 2016). In order to design synthetic RBS and enable rational control over protein expression levels, the “RBS calculator” was developed a decade ago (Salis et al., 2009; Salis, 2011). Experimental validations in *E. coli* showed that the method is accurate to within a factor of 2.3 over a range of 100,000-fold (*R* ^*2*^ = 0.54 on natural sequences and 0.84 on synthetic ones), correctly predicting the large effects of genetic context on identical RBS sequences that result in different protein levels. The tool was further expanded in a subsequent study (Espah Borujeni et al., 2017), where the N-terminal mRNA structures that need to be unfolded by the ribosome during translation initiation were precisely determined by designing and measuring expression levels of 27 mRNAs with N-terminal coding structures with varying positioning and energetics. The folding energetics of the N-terminal mRNA structures were determined to control translation rates only when the N-terminal mRNA structure overlaps with the ribosomal footprint, which extends 13 nucleotides past the start codon. By utilizing this improved quantification of the ribosomal footprint length, their biophysical model could more accurately predict the translation rates of 495 characterized mRNAs with diverse sequences and structures (*R* ^*2*^ = 0.78). The contribution of the Shine-Dalgarno sequence to protein expression was further comprehensively assessed and used to develop the tool “EMOPEC,” which can modulate the expression level of any *E. coli* gene by changing only a few bases (Bonde et al., 2016). Measured protein levels for 91% of the designed sequences were within twofold of the desired target levels, and predictions of these levels with RF regressors wastly outperformed RBS calculator with an *R* ^*2*^ of 0.89 compared to 0.44.

In eukaryotes, the nucleotide composition of the 5′ UTR changes across genes and species, with highly expressed genes in *S. cerevisiae* preferring A-rich and G-poor 5′ UTRs. The Kozak sequence, which helps to initiate translation in most mRNAs and occupies the first 6–9 nucleotides upstream of the START codon AUG, thus has the consensus 5′-WAMAMAA-3′ in yeast (Li et al., 2017a), whereas in humans this is 5′-GCCGCCRMC-3ʹ (Nakagawa et al., 2008). Measurement of protein abundance in 2,041 5′-UTR sequence variants, differing only in positions −10 to −1, showed that in yeast, key regulatory elements, including AUG sequence context, mRNA secondary structure, nucleosome occupancy and out-of-frame upstream AUGs conjointly modulate protein levels (Dvir et al., 2013). Based on these features, a predictive model could be developed that explains two-thirds of the expression variation. Recently, however, it was shown that also nucleotides upstream of the Kozak sequence are highly important (Li et al., 2017a). Ding and co. (Ding et al., 2018) synthesized libraries of random 5′ UTRs of 24 nucleotides and used a mathematical model accounting for strong epistatic interactions among bases to predict protein abundance. Then, by stepwise engineering the 5′ UTRs according to nucleotide sequence activity relationships (NuSAR), through repeated cycles of backbone design, directed screening, and model reconstruction, the predictive accuracy of the model was improved (*R* ^*2*^ = 0.71 vs. initial 0.60), resulting in strong 5′ UTRs with 5-fold higher protein abundance than the initial sequences. Similarly, a computational approach for predicting translation initiation rates, termed “yUTR calculator,” was developed using partial least-squares (PLS) regression and multiple predictive features, including presence of upstream AUGs (Decoene et al., 2018). This enabled the *de novo* design of 5′ UTRs with a diverse range of desired translation efficiencies, which were confirmed *in vivo*. Moreover, the importance of mRNA secondary structures in 5′ UTRs (Leppek et al., 2018) was also confirmed by inserting hairpin RNA structures into mRNA 5′ UTRs, which tuned expression levels by 100-fold by inhibiting translation (Weenink et al., 2018). This enables generating libraries with predicted expression outputs.

To facilitate deep learning of 5′ UTR function in yeast, a library of half a million 50 bp random 5′ UTRs was constructed and their activity assayed with growth selection experiments (Cuperus et al., 2017). A CNN model was generated that could accurately predict protein levels of both random and native sequences (*R* ^*2*^ = 0.62), and was used to evolve highly active 5′ UTRs that were experimentally confirmed to lead to higher protein expression rates than the starting sequences. Similarly, in human cells, polysome profiling of a library of 280,000 randomized 5′ UTRs was used to develop a CNN, termed “Optimus 5-Prime,” that could quantitatively capture the relationship between 5′ UTR sequences and their associated mean ribosome load (*R* ^*2*^ = 0.93 vs. 0.66 with k-mer based LR) (Sample et al., 2019). Combined with a genetic algorithm, the model was used to engineer new 5′ UTRs that accurately directed specified levels of ribosome loading, and also enabled finding disease-associated SNVs that affect ribosome loading and may represent a molecular basis for disease.

### 3′ Untranslated Region and Terminator

Regulatory motifs within the 3′ UTR and terminator region influence transcription termination, with 3′ UTR regulating polyadenylation, localization and stability (decay) of mRNA as well as translation efficiency (Barrett et al., 2012; Ren et al., 2017). The 3′ UTR contains both binding sites for regulatory proteins and microRNAs that can decrease gene expression by either inhibiting translation or directly causing mRNA degradation. It carries the A-rich ‘positioning’ element (consensus 5′-AAWAAA-3′ in yeast and 5′-AATAAA-3′ in humans) that directs addition of several hundred adenine residues called the poly(A) tail to the end of the mRNA transcript - the poly(A) site 5′-Y(A)n-3′, the TA-rich ‘efficiency’ element (most frequently 5′-TATWTA-3′) upstream of the positioning element and multiple T-rich sites (Guo and Sherman, 1996; Zhao et al., 1999; Curran et al., 2015). Based on these motifs, Curran and co. (Curran et al., 2015) developed a panel of short 35–70 bp synthetic terminators for modulating gene expression in yeast, the best of which resulted in a 3.7-fold increase in protein expression compared to that of the common CYC1 terminator. Further investigation of the effects of 13,000 synthetic 3′ end sequences on constitutive expression levels in yeast showed that the vast majority (∼90%) of strongly affecting mutations localized to a single positive TA-rich element, similar to the efficiency element (Vogel et al., 2010). Based on the strength of this element, dependent also on the GC content of the surrounding sequence, their classification model could explain a significant amount of measured expression variability in native 3′ end sequences (*r* = 0.41). Moreover, similarly as with promoters (Curran et al., 2014), Morse and co. (Morse et al., 2017) showed that terminator function can be modulated on the basis of predictions of nucleosome occupancy, with LR models highly predictive of protein output based on nucleosome occupancy scores (*R* ^*2*^ = 0.84). Designed terminators depleted of nucleosomes achieved an almost 4-fold higher net protein output than their original counterparts, with the main mode of action through increased termination efficiency, rather than half-life increases, suggesting a role in improved mRNA maturation.

Most genes express mRNAs with alternative polyadenylation sites at their 3′ ends (Tian and Manley, 2017), which were found to be remarkably heterogeneous across different yeast species. The polyadenylation pattern is determined by a broad degenerate sequence as well as local sequence reliant on poly(A) residues that can adopt secondary structures to recruit the polyadenylation machinery (Moqtaderi et al., 2013). In humans, alternative polyadenylation leads to multiple RNA isoforms derived from a single gene, and a CNN termed 'APARENT' was trained on isoform expression data from over three million reporters to infer alternative polyadenylation in synthetic and human 3′UTRs (Bogard et al., 2019). APARENT was shown to recognize known sequence motifs for polyadenylation, such as the positioning element, and also discover new ones, enabling the authors to engineer precisely defined polyadenylation signals and study disease-related genetic variants.

Bacterial transcription termination is known to occur *via* two distinct mechanisms: factor-dependent or factor-independent termination. The former relies on a regulatory protein Rho at Rho-dependent terminator sequences and is responsible for ∼20% of termination events in *E. coli* (Peters et al., 2009), whereas factor-independent termination accounts for the remaining ∼80% of transcription termination events and occurs at defined sequence regions known as “intrinsic terminators” that contain GC-rich regions (Roberts, 2019). Cambray and co. (Cambray et al., 2013) assembled a collection of 61 natural and synthetic intrinsic terminators that encode termination efficiencies across an 800-fold dynamic range in *E. coli* and, by simulating RNA folding, they found that secondary structures extending beyond the core terminator stem are likely to increase terminator activity. They developed linear sequence-function models that can accurately predict termination efficiencies (*r* = 0.67), further improving their performance by excluding terminators encoding the context-confounding structural elements (*r* = 0.9).

## Predicting Transcript and Protein Levels From Multiple Regulatory Parts

**FIGURE 3:**
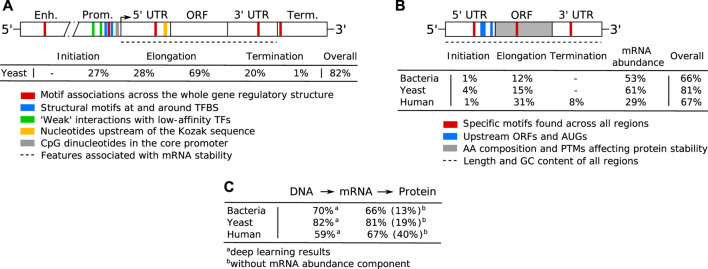
Quantifying gene expression and interpreting its regulatory grammar with machine learning. **(A)** Recently identified DNA regulatory elements predictive of mRNA abundance that expand the base knowledge depicted in Figure 1B. These include motif associations (Zrimec et al., 2020) (red), structural motifs (e.g. DNA shape, blue) (Zhou et al., 2015; Yang et al., 2017), weak interactions (de Boer et al., 2020) (green), nucleotides upstream of the Kozak sequence (Li et al., 2017a) (yellow), CpG dinucleotides (Agarwal and Shendure, 2020) (gray) and mRNA stability features (Neymotin et al., 2016; Cheng et al., 2017; Agarwal and Shendure, 2020; Zrimec et al., 2020) (dashed line, see text for details) identified in specific regions or across the whole gene regulatory structure. The table specifies the variation of mRNA abundance explained by DNA sequence and features using deep learning (Zrimec et al., 2020). Note that with alternative approaches, higher predictive values were obtained for certain regions in Table 2. **(B)** mRNA regulatory elements recently found to be predictive of protein abundance apart from features depicted in Figure 1C. These include specific motifs found across all regions (Li et al., 2019a; Eraslan et al., 2019b) (red), upstream ORFs (Vogel et al., 2010; Li et al., 2019a) and AUGs (Neymotin et al., 2016; Li et al., 2019a) (blue), AA composition (Vogel et al., 2010; Guimaraes et al., 2014) and post-translational modifications (PTMs) (Eraslan et al., 2019b) (gray) as well as lengths and GC content of all regions (Neymotin et al., 2016; Cheng et al., 2017; Li et al., 2019a) (dashed line). The table specifies the variation of protein abundance explained by mRNA levels and translational elements, using comparable shallow approaches in *E. coli* (Guimaraes et al., 2014), *S. cerevisiae* (Lahtvee et al., 2017) and *H. sapiens* (Vogel et al., 2010). Note that with alternative approaches, higher values were obtained for certain regions in Table 2. **(C)** Quantifying the central dogma of molecular biology with variance explained by mapping DNA to mRNA levels (Agarwal and Shendure, 2020; Zrimec et al., 2020) and mRNA levels to protein abundance (Vogel et al., 2010; Guimaraes et al., 2014; Lahtvee et al., 2017), using deep and shallow learning, respectively. Note that highly different modeling approaches were used.

The whole nucleotide sequence is involved in gene expression. When predicting the outcomes of transcription and translation, e.g. transcript and protein abundance, it is important to consider that many of the underlying steps in these processes are dependent on the outcome of the previous steps and some can occur in tandem (Watson et al., 2008) (Figures 1B,C). Each region of the gene and mRNA regulatory structures carries distinct regulatory signals that control the specific enzymatic interactions and thus encodes a significant amount of information related to mRNA (Shalem et al., 2015; Cheng et al., 2017; Cuperus et al., 2017; Zrimec et al., 2020) and protein levels (Vogel et al., 2010; Guimaraes et al., 2014; Lahtvee et al., 2017). Moreover, multiple lines of evidence support that the gene regulatory structure is a coevolving unit in both multicellular (Castillo-Davis et al., 2004; Wittkopp et al., 2004; Hahn, 2007; Wittkopp and Kalay, 2011; Arbiza et al., 2013; Naidoo et al., 2018; Washburn et al., 2019) and unicellular eukaryotes (Tirosh et al., 2009; Park et al., 2012; Chen et al., 2016; Zrimec et al., 2020), as genes display a coupling of coding and regulatory sequence evolution (Wittkopp et al., 2004; Tirosh et al., 2009; Zrimec et al., 2020) with approximately half of all functional variation found in non-coding regions (Hahn, 2007). However, although data from multiple regions was already used in prediction of mRNA and protein levels with shallow models (Vogel et al., 2010; Guimaraes et al., 2014; Lahtvee et al., 2017), predictions based on whole gene regulatory structures spanning multiple kilobases have started to emerge only recently, with the support of deep learning (Washburn et al., 2019; Zrimec et al., 2020). Accounting for multiple regions in ML models can lead to important observations, such as differentiating and quantifying the effects of separate vs. combined regions, and determining the DNA variables across the regions as well as their interactions, which affect predictions (Figure 3A).

DNNs are highly useful in learning the regulatory code of gene expression across regulatory structures. Despite hybrid CNN + RNN architectures outperforming them in terms of accuracy, CNNs work sufficiently well for this task (Yu and Koltun, 2015; Gupta and Rush, 2017; Strubell et al., 2017) and excel in learning rich higher-order sequence features that define the *cis*-regulatory grammar (Siggers and Gordân, 2014; Zeng et al., 2016). Systematic analyses of network properties, such as CNN kernel size, number of kernels and number of layers as well as pooling designs (pooling layers between connected CNNs), have exemplified how DNNs decode the regulatory grammar in sequence-based learning tasks (Trabelsi et al., 2019; Zeng et al., 2016; Koo and Eddy, 2019) (Figure 2D). In a multilayer DNN, the initial one to two layers capture information on single motif occurrence, with the first layer potentially learning partial motif representations. This can be useful in complicated tasks, such as learning DNA regulatory grammar, because a wider array of representations can be combinatorially constructed from partial representations to capture the rich array of biologically important sequence patterns *in vivo* (Siggers and Gordân, 2014; Zrimec et al., 2020). Successive layers (e.g. Third layer) then learn to recognize motif interactions (i.e. associations in predicting the target variable) across the regulatory structure (Zeng et al., 2016; Zrimec et al., 2020). The extent to which sequence motif representations are learned by first layer kernels is influenced by kernel size and pooling, which enforce a spatial information bottleneck either from the sequence to the CNN or between successive CNN layers, respectively. For instance, large max-pooling (≥10) was shown to force kernels to learn whole motifs, whereas CNNs that employ a low max-pool size (≤4) capture partial motifs (Koo and Eddy, 2019). Similarly, the size of successive convolutional kernels can also affect the ability to assemble whole motifs in deeper layers. Moreover, the number of kernels in the first layer sets a hard constraint on the number of different sequence patterns that can be detected (Koo and Eddy, 2019). Since the scope of initial characterized sequence features limits the range and complexity of grammar representations that can be built downstream, this parameter was generally found to have the greatest impact on CNN performance (Zeng et al., 2016). Therefore, in contrast to learning tasks where the main features are simple, such as occurrence of a PWM-like motif in TFBS prediction, using multiple parts of the regulatory structures requires deeper and more complex architectures that learn distributed motif representations to address the more complex sequence patterns (Koo and Eddy, 2019; Trabelsi et al., 2019) (Figure 2D).

### Predicting messenger RNA Levels From Nucleotide Sequence

Despite the importance of the whole gene regulatory structure in gene expression, very few combinations of regulatory elements have been tested and their functional interactions remain poorly explored. To estimate the contribution of individual regulatory parts in gene expression, a combinatorial library of regulatory elements including different enhancers, core promoters, 5′ UTRs and transcription terminators was constructed in *S. cerevisiae* (Dhillon et al., 2020). A strong interaction was found between enhancers and promoters, showing that, while enhancers initiate gene expression, core promoters modulate the levels of enhancer-mediated expression and can positively or negatively affect expression from even the strongest enhancers. Interestingly, principal component analysis indicated that enhancer and promoter function can be explained by a single principal component. Espinar and co. (Espinar et al., 2018) tested if promoters and coding regions can be understood in isolation, or if they interact, by measuring mRNA levels for 10,000 constructs. The strength of cotranslational regulation on mRNA levels from either inducible or constitutive promoter architecture was explored using LR, where a novel mechanism for co-regulation with inducible promoters was identified (RNA helicase Dbp2), whereas with constitutive promoters, most of the information on mRNA levels was found in the coding region and not in the promoter (Table 3). Neymotin and co. (Neymotin et al., 2016) analyzed both coding regions and their interactions with other *cis*-regulatory variables in mRNA transcripts that affect mRNA degradations rates (which in turn affect overall mRNA abundance) using multiple LR. Multiple transcript properties were significantly associated with variation in mRNA degradation rates, including transcript length, ribosome density, CUB and GC content at the third codon position, and a model incorporating these properties explained ∼50% of the genome-wide variance. A similar quantitative model based on functional mRNA sequence features explained 59% of the half-life variation between genes, predicting half-life at a median relative error of 30% (Cheng et al., 2017). mRNA sequence features found to most strongly affect mRNA stability included CUB (*R* ^*2*^ = 0.55), destabilizing 3′ UTR motifs, upstream AUG codons, UTR lengths and GC content.

**TABLE 3:** Overview of studies modeling transcript and protein-abundance related properties from combined regulatory and coding regions. Highest achieved or average scores are reported, on test sets where applicable, and include area under the receiver operating characteristic curve (AUC), area under the precision recall curve (AUPRC), the coefficient of variation (*R* ^*2*^) and Spearman's correlation coefficient (*ρ*).

Ref.	Strategy	Region	Target var.	Explan. vars.	Method	Score	Organism
(Espinar et al., 2018)	Shallow	Promoter, coding	mRNA abundance	DNA sequence features	LR	*R* ^*2*^ = 0.64	Yeast
(Neymotin et al., 2016)	Shallow	mRNA transcript	mRNA stability (degradation rates)	mRNA features	Multiple LR	*R* ^*2*^ = 0.50	Yeast
(Cheng et al., 2017)	Shallow	mRNA transcript	mRNA stability (half-life)	mRNA features	Multivariate LR	*R* ^*2*^ = 0.59	Yeast
(Zhou et al., 2018a)	Deep	Whole gene regulatory structure	mRNA abundance	DNA sequence	CNN + L2-regularized LR	AUC = 0.82	Human
(Zrimec et al., 2020)	Deep	Whole gene regulatory structure	mRNA abundance	DNA sequence and features	CNN regression	*R* ^*2*^ = 0.82, 0.70, 0.42, respectively	Yeast, *E. coli*, human
(Agarwal and Shendure, 2020)	Deep	Promoter, coding	mRNA abundance	DNA sequence and features	CNN regression	*R* ^*2*^ = 0.59	Human
(Zhang et al., 2020)	Deep	Whole gene regulatory structure	mRNA abundance	DNA sequence	ResNet regression	*ρ* = 0.80	Human
(Guimaraes et al., 2014)	Shallow	mRNA transcript	Protein abundance	mRNA features	PLS regression	*R* ^*2*^ = 0.66	*E. coli*
(Lahtvee et al., 2017)	Shallow	mRNA transcript	Protein abundance	mRNA features	MARS nonlinear regression	*R* ^*2*^ = 0.81	Yeast
(Vogel et al., 2010)	Shallow	mRNA transcript	Protein abundance	mRNA features	MARS nonlinear regression	*R* ^*2*^ = 0.67	Human
(Li et al., 2019a)	Shallow	mRNA transcript	Translation rates	mRNA features	Multivariate LR	*R* ^*2*^ = 0.81, 0.42, respectively	Yeast, human
(Terai and Asai, 2020)	Shallow	mRNA transcript	Protein abundance	mRNA features of translation initiation	RF regression	*ρ* = 0.76	*E. coli*
(Li et al., 2017b)	Shallow	mRNA transcript	Translation rates	mRNA features	Bayesian model	*R* ^*2*^ = 0.20 (TR_mD_,; 0.80 (TR_mIND_)	Yeast
(Eraslan et al., 2019b)	Shallow	mRNA transcript	Protein-to-RNA ratio	mRNA sequence and features	Multivariate LR	*R* ^*2*^ = 0.62	Human
(Zhang et al., 2017b)	Deep	mRNA transcript	Translation initiation sites	mRNA sequence	CNN + RNN classification	AUPRC = 0.62	Human
(Zhang et al., 2017c)	Deep	mRNA transcript	Translation elongation dynamics	mRNA sequence	CNN classification	AUC = 0.88, 0.83, respectively	Yeast, human

Recently, deep learning was applied on over 20,000 mRNA datasets in seven model organisms that included bacteria, yeast and human, to examine how individual coding and non-coding regions of the gene regulatory structure interact and contribute to mRNA abundance (Zrimec et al., 2020). The CNN-based approach, termed “DeepExpression,” could predict the variation of transcript levels directly from DNA sequence in all organisms, with up to 82 and 70% achieved in *S. cerevisiae* and *E. coli*, respectively, outperforming shallow methods by over 13%. Apart from the DNA sequence, CUB and features associated with mRNA stability, including lengths of UTRs and open reading frames (ORFs), UTR GC content and GC content at each codon position (Neymotin et al., 2016; Cheng et al., 2017), were found to increase the predictive power of the models. Compared to single interpreted DNA motifs, motif associations could explain a much larger portion of the dynamic range of mRNA levels (84 vs. 57%), suggesting that instead of single motifs and regions, the entire gene regulatory structure with specific combinations of regulatory elements defines gene expression levels (Figure 3A). This was also supported by observations of co-evolution among coding and non-coding regions across 14 related yeast species. With similar objectives, Agarwal and Schendure (Agarwal and Shendure, 2020) developed “Xpresso,” which could explain 59 and 71% of variation in steady-state mRNA levels in human and mouse, respectively, based only on promoter sequences and explanatory features associated with mRNA stability. They showed that Xpresso more than doubles the accuracy of alternative sequence-based models and model interpretation revealed that promoter-proximal CpG dinucleotides strongly predict transcriptional activity.

To predict the tissue-specific transcriptional effects of genome variation, including rare or unseen mutations, Zhou and co. (Zhou et al., 2018a) developed a DNN–based framework termed “ExPecto.” Using ExPecto to profile over 140 million promoter-proximal mutations, the authors characterized the regulatory mutation space for human RNAP II–transcribed genes, which enables probing of evolutionary constraints on gene expression and *ab initio* prediction of mutation disease effects. A similar model was constructed using residual networks (ResNets), which are multilayer CNNs that utilize *skip connections* to jump over some layers (He et al., 2016), termed “ExpResNet” (Zhang et al., 2020). By utilizing almost 100 kb of sequence around each gene”s TSS, ExpResNet outperformed existing models, including ExPecto (*ρ =* 0.80 vs. 0.75), across four tested tissues. Interestingly, by comparing the performance achieved with different input sequence sizes, we can observe that the majority of regulatory information in humans is constrained to ∼10 kb of regulatory structure around the TSS (*ρ* = 0.77, 0.79, 0.80 with 10, 40 and 95 kb, respectively), likely since this is sufficient for the majority of genes, whereas enhancers outside of this region are gene-specific and positioned highly variably.

### Predicting Protein Abundance From mRNA Sequence

In multiple organisms, protein levels at steady state are primarily determined by mRNA levels, where up to ∼85% of the variation of protein expression can be attributed to mRNA transcription rather than protein translation (Schwanhäusser et al., 2011; Csárdi et al., 2015; Liu et al., 2016). Nevertheless, the spatial and temporal variations of mRNAs and the local availability of resources for protein biosynthesis strongly influence the relationship between protein levels and their transcripts (Liu et al., 2016). Thus, in many scenarios, transcript levels by themselves are not sufficient to predict protein levels and multiple other mRNA-related properties and processes affect translation and define the final gene expression levels. It was also shown that, due to translation rates per mRNA molecule being positively correlated with mRNA abundance, protein levels do not scale linearly with mRNA levels, but instead scale with the abundance of mRNA raised to the power of an “amplification exponent” (Csárdi et al., 2015). Li and co. (Li et al., 2017b) proposed that, to quantify translational control, the translation rate must be decomposed mathematically into two components: one that is dependent on mRNA abundance (TR_mD_), defining also the amplification exponent, and one that is not (TR_mIND_). In yeast, TR_mD_ represented ∼20% of the variance in translation, whereas TR_mIND_ constituted the remaining ∼80% of the variance in translation. The components were also preferentially determined by different mRNA sequence features: TR_mIND_ by the length of the ORF and TR_mD_ by a ∼60 nt element spanining the initiating AUG and by CUB, implying that these components are under different evolutionary selective pressures.

Quantification of absolute protein and mRNA abundances for over 1,025 genes from the human Daoy medulloblastoma cell line showed that the combined contribution of mRNA levels and sequence features can explain ⅔ of protein abundance variation at steady state (Vogel et al., 2010) (Figure 3B). Using multivariate adaptive regression splines (MARS), a nonlinear regression technique, the variation in protein abundance was primarily explained by translation elongation factors (31%), with an impact similar to that of mRNA abundance (29%). The strongest individual correlates of protein levels were translation and degradation-related features including mRNA sequence length, AA properties, upstream ORFs and 5′ UTR secondary structures. Interestingly, characteristics of the 3′ UTR explained a larger proportion of protein abundance variation (8%) than characteristics of the 5′ UTR (1%). A similar analysis performed with 824 genes in *E. coli*, which used PLS regression and over 100 mRNA sequence features, also derived a model that explained ⅔ of the total variation of protein abundance (Guimaraes et al., 2014). The model suggests that protein abundance is primarily determined by the transcript level (53%) and by effectors of translation elongation (12%), which included both CUB and specific AA composition, whereas only a small fraction of the variation is explained by translation initiation (1%). Lahtvee and co. (Lahtvee et al., 2017). measured absolute abundances of 5,354 mRNAs and 2,198 proteins in yeast under different environmental conditions, showing that the overall correlation between mRNA and protein abundances across all conditions is much higher for a subset of 202 differentially expressed proteins than all of them (avg. *r* = 0.88 vs. 046). On a subset of 1,117 proteins, for which translation efficiencies were calculated, MARS detected that mRNA abundance and translation elongation were the dominant factors controlling protein synthesis, explaining 61 and 15% of its variance, with only a small fraction (4%) explained by translation initiation (Figure 3B).

On the other hand, multiple recent studies show that general mRNA features control a much larger fraction of the variance in translation rates or protein abundance than previously realized. For instance, Li and co. (Li et al., 2019a) quantified the contributions of mRNA sequence features to predicting translation rates using LR across multiple organisms, including yeast and human, where they specified 81 and 42% of the variance in translation rates, respectively. The identified informative mRNA features included similar ones as found in previous studies: 5′ UTR secondary structures, nucleotides flanking AUG, upstream ORFs, ORF length and CUB (Vogel et al., 2010; Neymotin et al., 2016; Cheng et al., 2017).

Eraslan and co. (Eraslan et al., 2019b) also showed that a large fraction of protein abundance variation can be predicted from mRNA sequence in humans, by analyzing 11,575 proteins across 29 human tissues using matched transcriptomes and proteomes. Their initial LR model explained on average 22% of the variance from sequence alone, and by including additional experimentally characterized interactions and modifications, including mRNA methylation (Zhao et al., 2017), miRNA and RBP binding sites (Mayr, 2017) and post-translational modifications (Millar et al., 2019), the explained variance increased to 62%. Their findings support much of the previously identified mRNA regulatory elements and also uncover new sequence motifs across the whole transcript. Importantly, they also developed a new metric of codon optimality, termed “Protein‐to‐mRNA adaptation index” that captures the effects of codon frequency on protein synthesis and degradation. Terai and Asai (Terai and Asai, 2020) evaluated six types of structural features in *E. coli*, including mRNA accessibility, which is the probability that a given region around the start codon has no base-paired nucleotides. When calculated by a log-linear model, accessibility showed the highest correlation with protein abundance. This was significantly higher than the widely used minimum free energy (*ρ* = 0.71 vs. 0.55), and combining it with activity of the Shine-Dalgarno sequence yielded a highly accurate method for predicting protein abundance (*ρ* = 0.76). Moreover, similarly as in eukaryotes, secondary structures in bacterial mRNAs were shown to be highly important for protein production and to generally limit translation initiation in a large-scale assay involving 244,000 designed sequences with varying features (Cambray et al., 2018).

Deep learning was recently applied to prediction of translation initiation sites in a method termed “TITER” (Zhang et al., 2017b), using HTS data quantitatively profiling initiating ribosomes (QTI-seq) at single-nucleotide resolution (Gao et al., 2015). Using a hybrid CNN + RNN approach, TITER integrates the prior preference of TIS codon composition with translation initiation features extracted from the surrounding sequence to greatly outperform other state-of-the-art methods in predicting the initiation sites. The method captures the sequence motifs of different start codons, including a Kozak sequence-like motif for AUG, and quantifies mutational effects on translation initiation. Another DNN framework, termed “ROSE” was used to analyze translation elongation dynamics in both human and yeast *via* ribosome stalling, which is manifested by the local accumulation of ribosomes at specific codon positions of mRNA (Zhang et al., 2017c). ROSE estimates the probability of a ribosome stalling event occurring at each genomic location, achieving higher prediction accuracy than conventional prediction models such as gkm-SVM with AUC increases by up to 18.4%.

## Discussion

As can be surmised from the presented ML results (Table1, Table 2, and Table 3), deep methods frequently outperform shallow ones, and we outline the main advantages, disadvantages and challenges of these approaches in Table 4. The capability of DNNs to more accurately recapitulate experimental data stems mainly from their ability to extract information directly from the raw input nucleotide sequences, automatically learning regulatory grammar (Figures 2B,D), which boosts predictive accuracy (Zrimec et al., 2020). However, although multiple different methods exist for interpreting deep methods, many are a work in progress and no explicit solutions currently exist to benchmark these methods or to combine the findings into more complete and coherent interpretations (Azodi et al., 2020). Nevertheless, ML in general lowers the entry barrier to development of new models and saves research time by abstracting mathematical details (Eraslan et al., 2019a), though this has also been used to criticize such approaches, as they rely on data driven instead of hypothesis driven modeling (Barshai et al., 2020). An important limitation of all ML methods is their dependence on accurately labeled data, since they cannot achieve higher accuracy than that allowed by the noise inherent to the given target labels (Li et al., 2019b; Barshai et al., 2020). For instance, *in vivo* measurements, such as those produced by ChIP-seq, ATAC-seq and DNAse-seq, are prone to experimental noise and technological artifacts and subject to the complexity of the cellular environment, affected by chromatin structure and nucleosome positioning, thus concealing the full picture of DBP-DNA interactions. Alternatively, *in vitro* methods, such as PBMs, HT-SELEX and BunDLE-seq, can capture purely direct protein-nucleic acid interactions or cooperative binding of specific factors and allow sampling of the full spectrum of binding sites (Barshai et al., 2020). Fortunately, novel computational methods allow researchers to easily estimate the noise-constrained upper bound of ML regression model performance (Li et al., 2019b).

**TABLE 4:** The current advantages, disadvantages and further challenges of machine learning methods in genetics and genomics.

	Deep methods	Shallow methods
Advantages	Lower entry barrier to develop new models and save research time by abstracting mathematical details (Eraslan et al., 2019a)	
	Scale effectively with data and support use of latest computational and technological advances, including large genomic datasets and results of HTS technologies (Barshai et al., 2020)	Classic statistical models are better characterized mathematically and some ML algorithms are easier to understand and explain (Hastie et al., 2013)
	Ability to automatically learn features from raw input data and unlock an additional level of information from it (Barshai et al., 2020; Zrimec et al., 2020)	Less computationally expensive and faster to train leading to more iterations and testing of different techniques in a shorter period of time
	Ability to learn and approximate complex functions without prior assumptions, frequently achieving improved predictive power (Barshai et al., 2020)	Possibility to train on much smaller datasets (e.g. hundreds of examples vs. thousands or more with deep learning) (Playe and Stoven, 2020; Zrimec et al., 2020)
	Capability to integrate multiple pre-processing steps into a single end-to-end model (Eraslan et al., 2019a)	Can be easier to interpret due to inherently interpretable structure and direct feature engineering/selection (Figures 2A,C) (Azodi et al., 2020)
	Ability to effectively model multimodal data (Eraslan et al., 2019a)	Usually a small number of hyperparameters (Hastie et al., 2013)
	Highly useful as experiment simulators due to the ability to generalize over an experimental dataset (Barshai et al., 2020)	Useful for proof-of-principle and initial model or parameter testing using only numerical variables
	Easily adaptable to different domains and applications, with transfer learning on pre-trained deep networks accelerating training and improving performance	—
Disadvantages	Dependence on accurately labeled data: cannot achieve higher accuracy than that allowed by the noise inherent to the given experimental target labels (Li et al., 2019b; Barshai et al., 2020)	
	Data driven instead of hypothesis driven modeling (Barshai et al., 2020)	
	Dependence on large amounts of data (at least thousands of training examples) and specialized computational resources (e.g. GPUs)	Dependence on feature engineering
	Potential problems with generalizability, as can be overfit to the experiment rather than biological function (Barshai et al., 2020)	Many different algorithms each with its own advantages and disadvantages can be daunting and require extensive specialized study (Hastie et al., 2013)
	Potential lack of model interpretability (Zou et al., 2019; Barshai et al., 2020)	Cannot unlock information directly from nucleotide sequence (Azodi et al., 2020; Zrimec et al., 2020)
Challenges	Methods to interpret heterogeneous multi-omic and highly dimensional data (Azodi et al., 2020)	
	Methods and high quality datasets to benchmark existing and new interpretation strategies (Azodi et al., 2020)	
	Methods to join findings from multiple interpretation strategies into more complete and coherent interpretations of both models (Azodi et al., 2020) and the studied molecular phenomena	
	Making interpretable ML more accessible to biologists by further lowering the entry barriers and requirements of computational knowledge (Azodi et al., 2020)	

Despite the knowledge that whole regulatory structures are involved in gene expression, the majority of approaches still focus only on single regulatory or coding regions. For instance, with mRNA abundance prediction, the contribution of the separate parts of the gene regulatory structure has been quantified only in yeast (Zrimec et al., 2020) (Figure 3A). The results across the remaining studies are highly variable, likely due to using very different methods and protocols (Table 2 and Table 3). The trend of using whole regulatory structures is however more common with protein abundance prediction, where, apart from mRNA abundance, also the parts involved in translational processing have been quantified across all three major model organisms (Figure 3B). Nevertheless, both the fact that these studies were performed using classical shallow models as well as results from other studies suggest that there is potential for improvement. For instance, results from multiple studies focusing on individual regions show that a much higher amount of information can be extracted from these regions [Table 2: e.g. 62% of protein abundance variation explained from yeast 5′ UTRs with DNNs (Decoene et al., 2018)] than was achieved with shallow learning on whole mRNAs in Figure 3B. Based on other results, we can also presume that it is possible to not only further boost predictive performance but also uncover new mRNA regulatory grammar.

Pooling the highest-scoring results across organisms in an information-centric view of the central dogma of molecular biology (Figure 3C) suggests that about ⅔ of the variation of mRNA and protein levels can be explained from DNA sequence. Unequal approaches were employed however, with deep learning used only with mRNA abundance modeling. Here, the lower results with *H. sapiens* might be a result of accounting for only promoter regions and mRNA stability-associated features in the model (Agarwal and Shendure, 2020), though our own analysis had shown that these stability features alone can explain 38% of the mRNA abundance variation in yeast (Zrimec et al., 2020). Interestingly, by omitting the mRNA abundance component from protein abundance predictions, we can observe the possibility of an increasing trend of explained variance with increasing organism complexity (Figure 3C: 13–40% from bacteria to human). This would indicate that mRNAs of multicellular eukaryotes carry more regulatory information involved in translation than those of unicellular eukaryotes and prokaryotes. It might also reflect the fact that gene expression regulation is more intricate in multicellular organisms due to the multiple additional regulatory processes that control expression of a much more complex set of biomolecules and phenotypes than in unicellular organisms (Benelli et al., 2016).

Regulatory information seems to be localized *around* the gene, as multiple studies show that the region spanning <10 kb around the TSS has the largest measurable effect on gene expression, likely as the majority of regulatory signals are clustered in this region in most genes and organisms (Agarwal and Shendure, 2020; Ansariola et al., 2020; Zrimec et al., 2020). Enhancers on the other hand are highly variably spaced and act in a gene-specific manner, which makes them much harder to recognize, and also requires processing enormous sizes of input sequences (e.g. >100 kb upstream of genes in human data) that require more training resources. Therefore, the true effect of such regions is still hard to decipher. Procedures handling larger input sequence sizes or whole genomes will likely lead to improved analysis and quantification of the contributions of enhancers to gene expression control, in relation to other parts of the regulatory structure (Singh et al., 2019; Tang et al., 2020). Another potential trend is building DNNs using biophysical (Tareen and Kinney, 2019) or physicochemical properties (Yang et al., 2017; Liu et al., 2020), as deep models trained on these features might uncover novel patterns in data and lead to improved understanding of the physicochemical principles of protein-nucleic acid regulatory interactions, as well as aid model interpretability. Other novel approaches include: 1) modifying DNN properties to improve recovery of biologically meaningful motif representations (Koo and Ploenzke, 2021), 2) transformer networks (Devlin et al., 2018) and attention mechanisms (Vaswani et al., 2017), widely used in protein sequence modeling (Jurtz et al., 2017; Rao et al., 2019; Vig et al., 2020; Repecka et al., 2021), 3) graph convolutional neural networks, a class of DNNs that can work directly on graphs and take advantage of their structural information, with the potential to give us great insights if we can reframe genomics problems as graphs (Cranmer et al., 2020; Strokach et al., 2020), and 4) generative modeling (Foster, 2019), which may help exploit current knowledge in designing synthetic sequences with desired properties (Killoran et al., 2017; Wang Y. et al., 2020). With the latter, unsupervized training is used with approaches including: 1) autoencoders, which learn efficient representations of the training data, typically for dimensionality reduction (Way and Greene, 2018) or feature selection (Xie et al., 2017), 2) generative adversarial networks, which learn to generate new data with the same statistics as the training set (Wang Y. et al., 2020; Repecka et al., 2021), and 3) deep belief networks, which learn to probabilistically reconstruct their inputs, acting as feature detectors, and can be further trained with supervision to build efficient classifiers (Bu et al., 2017). Moreover, the advent of single-cell HTS technologies such as single-cell RNA-seq will offer many novel research opportunities, including modeling of cell-type or cell-state specific enhancer or TFBS activations and chromatin changes (Angermueller et al., 2017; Gustafsson et al., 2020; Kawaguchi et al., 2021).

To conclude, the application of ML in genomics has augmented experimental methods and facilitated accumulating a vast amount of knowledge on gene expression regulation. DNNs, due to their ability to learn biologically relevant information directly from sequence, while performing similarly to or better than classical approaches, are the method of choice for quantifying gene expression and interpreting the predictive features hidden in nucleotide sequence data. DNN-isolated features can be as predictive as models relying on experimental ChIP-seq data (Agarwal and Shendure, 2020), suggesting that current computational approaches are achieving a level of accuracy that might soon allow substituting wet-lab HTS experiments with fully computational pipelines (Keilwagen et al., 2019). Such pipelines can also become indispensable for analysis of human disease-associated regulatory mutations, identifying clinically relevant noncoding variants and expression perturbations, grouping patients in drug treatment trials, disease subtyping as well as personalized treatment (Zhou et al., 2018a; Dagogo-Jack and Shaw, 2018). Since controlling the expression of genes is also one of the key challenges of synthetic biology, the computational models represent excellent starting points in procedures to predictably design regulatory sequences, control protein expression and fine-tune biosynthetic pathways in both prokaryotic and eukaryotic systems (Nielsen and Keasling, 2016; de Jongh et al., 2020; Wang H. et al., 2020).

For readers willing to learn and apply some of the discussed ML approaches, many excellent resources exist, including: 1) specialized packages for model development and interpretation, such as “DragoNN” (https://kundajelab.github.io/dragonn/) (Movva et al., 2019) “Janggu” (https://github.com/BIMSBbioinfo/janggu) (Kopp et al., 2020) and “Pysster” (https://github.com/budach/pysster) (Budach and Marsico, 2018), 2) repositories of trained models, such as “Kipoi” (https://kipoi.org/) (Avsec et al., 2019), 3) other genomics tutorials and code examples (https://github.com/vanessajurtz/lasagne4bio) (Jurtz et al., 2017), as well as 4) resources with a much broader scope than mere genomics, including online courses (https://www.coursera.org/specializations/deep-learning) and books (https://github.com/ageron/handson-ml2) (Géron, 2019).

## Abbreviations

AA, amino acid; AUC, area under the receiver operating characteristic curve; AUPRC, area under the precision recall curve; biLSTM, bidirectional long short-term memory; BunDLE-seq, binding to designed library, extracting, and sequencing; CAI, codon adaptation index; ChIP-seq, chromatin immunoprecipitation sequencing; CNN, convolutional neural network; CUB, codon usage bias; DBP, DNA-binding protein; DHS, DNase I hypersensitive site; DNase-seq, DNase I hypersensitive sites sequencing; DNN, deep neural network; dsDNA, double-stranded DNA; GM, geometric mean of precision and recall; HTS, high-throughput sequencing (technology); LR, linear regression; MARS, multivariate adaptive regression splines; MCC, Matthews correlation coefficient; ML, machine learning; mRNA, messenger RNA; NN, feedforward neural network; NuSAR, nucleotide sequence activity relationships; OLS, ordinary least squares (regression); ORF, open reading frame; PBM, protein binding microarray; PIC, (eukaryotic) preinitiation complex; PLS, partial least squares (regression); pre-mRNA, precursor mRNA; PTM, post-translational modifications; PWM, position weight matrix; RBP, RNA-binding protein; RBS, ribosome binding site; RF, random forest; RNAP, RNA polymerase; RNN, recurrent neural network; SELEX, systematic evolution of ligands by exponential enrichment; SVM, support vector machine; SNV, single nucleotide variant; ssDNA, single-stranded DNA; TF, transcription factors; TFBS, TF binding site; TIF, transcription initiation frequency; TSS, transcription start site; UTR, untranslated region.
